# Repurposing cabozantinib with therapeutic potential in KIT-driven t(8;21) acute myeloid leukaemias

**DOI:** 10.1038/s41417-021-00329-1

**Published:** 2021-04-08

**Authors:** Kuan-Wei Su, Da-Liang Ou, Yu-Hsuan Fu, Hwei-Fang Tien, Hsin-An Hou, Liang-In Lin

**Affiliations:** 1 Department of Clinical Laboratory Sciences and Medical Biotechnology, National Taiwan University, Taipei, Taiwan; 2 Department of Oncology, National Taiwan University, Taipei, Taiwan; 3 Department of Internal Medicine, National Taiwan University Hospital, Taipei, Taiwan; 4 Department of Laboratory Medicine, National Taiwan University Hospital, Taipei, Taiwan

**Keywords:** Targeted therapies, Drug development

## Abstract

Cabozantinib is an orally available, multi-target tyrosine kinase inhibitor approved for the treatment of several solid tumours and known to inhibit KIT tyrosine kinase. In acute myeloid leukaemia (AML), aberrant KIT tyrosine kinase often coexists with t(8;21) to drive leukaemogenesis. Here we evaluated the potential therapeutic effect of cabozantinib on a selected AML subtype characterised by t(8;21) coupled with *KIT* mutation. Cabozantinib exerted substantial cytotoxicity in Kasumi-1 cells with an IC_50_ of 88.06 ± 4.32 nM, which was well within clinically achievable plasma levels. The suppression of KIT phosphorylation and its downstream signals, including AKT/mTOR, STAT3, and ERK1/2, was elicited by cabozantinib treatment and associated with subsequent alterations of cell cycle- and apoptosis-related molecules. Cabozantinib also disrupted the synthesis of an AML1-ETO fusion protein in a dose- and time-dependent manner. In a mouse xenograft model, cabozantinib suppressed tumourigenesis at 10 mg/kg and significantly prolonged survival of the mice. Further RNA-sequencing analysis revealed that mTOR-mediated signalling pathways were substantially inactivated by cabozantinib treatment, causing the downregulation of ribosome biogenesis and glycolysis, along with myeloid leukocyte activation. We suggest that cabozantinib may be effective in the treatment of AML with t(8;21) and *KIT* mutation. Relevant clinical trials are warranted.

## Introduction

Differentiation arrest and uncontrollable proliferation of myeloid progenitor cells induce acute myeloid leukaemia (AML), a genetically heterogeneous clonal malignancy [1]. The t(8; 21) (q22; q22) reciprocal chromosome translocation is one of the most common chromosomal abnormalities in AML, accounting for ~13% of all AML cases and up to 40% of AML M2 subtype (FAB classification) [2, 3]. Chromosome translocation t(8;21) generates an aberrant fusion protein RUNX1-RUNX1T1 (AML1-ETO), which causes differentiation blockage [4].

AML1-ETO alone fails to induce leukaemogenesis; an additional coinciding mutation such as KIT, FLT3, JAK2, RAS, and PDGFR, is needed for full-blown leukaemia [5]. The *KIT* mutation frequency in t(8;21) leukaemia can be as high as 50% [6]; the coexistence of the two alterations is linked to a higher incidence of relapse after intensive chemotherapy and associated with adverse prognosis [3, 7].

Cabozantinib (XL-184) is an ATP-competitive multi-kinase inhibitor targeting MET, VEGFR2, RET, KIT, TIE-2, and FLT3, all of which are involved in cancer pathophysiology [8]. The US Food and Drug Administration (FDA) approved cabozantinib as a treatment for progressive metastatic medullar thyroid cancer, advanced renal cell carcinoma, and advanced hepatocellular carcinomas [9–11]. We utillised a large-scale integrated cancer cell line compound database, Genomics of Drug Sensitivity in Cancer (GDSC, https://www.cancerrxgene.org/), to assess the activity of cabozantinib against haematological malignancies. Among these malignancies, cabozantinib was especially effective against AML, according to low IC_50_ values (Supplementary Fig. 1A–C). Our previous sensitivity assessment of leukaemic cell lines (MV4-11, MOLM13, OCI-AML3, THP-1, RS4;11, K562, U937, and HL60) indicated that nanomolar cabozantinib was selectively cytotoxic in leukaemic cells with *FLT3*-ITD [12]. Subsequently, Klaeger also got similar results [13]. Fathi et al. [14] observed that cabozantinib was well tolerated in AML patients with *FLT3*-ITD and effectively inhibited the *FLT3*-mutated AML, demonstrating the feasibility of cabozantinib for AML treatment.

Based on the pharmacological potential of cabozantinib, we investigated the anti-leukaemia effect of cabozantinib on another subtype AML harbouring both *KIT* mutation and t(8;21).

## Materials and methods

### Cell culture and chemicals

Three human AML cell lines, Kasumi-1 (ATCC, Manassas, VA) with t(8;21) and heterozygous *KIT* N822K mutation, SKNO-1 (JCRB, Osaka, Japan) with t(8;21) and homozygous *KIT* N822K mutation, and OCI-AML3 (DSMZ, Brauschweig, Germany) with *NPM1* mutation and *DNMT3A* mutation, were used in this study. Kasumi-1 cells were maintained in RPMI 1640 medium (ThermoFisher Scientific/GIBCO, Waltham, MA) containing 20% fetal bovine serum (FBS), SKNO-1 cells in RPMI 1640 medium with 10% FBS containing 10 ng/ml granulocyte-macrophage colony-stimulating factor (GM-CSF; R&D systems, Minneapolis, MN), and OCI-AML3 cells in αMEM medium (GIBCO) containing 20% FBS. Cultures were grown at 37 °C in humidified atmosphere containing 5% CO_2_. These cell lines were authenticated (16-Markers STR) by the Food Industry Research and Development Institute, Hsinchu, Taiwan. Their genetic profiles were identical to reported information.

Cabozantinib-malate was purchased from Selleck Chemicals (Houston, TX) and dissolved in DMSO for storage at −20 °C. Cycloheximide (CHX), MG-132, Z-VAD-FMK, and puromycin were purchased from TargetMol (Boston, MA), Tocris Bioscience (Abingdon, UK), Abcam (Cambridge, MA), and GIBCO, respectively.

### Cellular growth assays

After 72-h drug treatments, cell viability was assessed with CellTiter 96 AQueous One Solution Cell Proliferation Assay (MTS assay; Promega, Madison, WI), according to the manufacturer’s instructions. The half-maximal inhibitory concentration (IC_50_) was calculated using the CalcuSyn software (Biosoft, Cambridge, UK).

Trypan blue exclusion assay was used for evaluating cell proliferation curves after drug treatment. Six-well plates were seeded with 2 × 10^5^ cells/well. After indicated treatment periods, the number of viable cells was determined using trypan blue dye (Merck, Kenilworth, NJ).

### Glucose uptake assay

Six-well plates were seeded with 1 × 10^6^ cells/well; control wells were loaded with cell-free culture medium for initial glucose measurements. After 16 h incubation, double-concentrated drug solutions were added for 24 h drug treatments. Then, plates were centrifuged at 400 × *g*, 4 °C, for 5 min. Supernatants were collected and mixed with equal volume of 1X Reaction buffer for measuring glucose with ACCU-CHEK glucose metre (Accu-Chek Performa, Roche Diagnostics, Switzerland). Glucose uptake was calculated by subtracting glucose values measured after 24-h drug treatment from initial glucose values: Glucose uptake = concentration of glucose_cell-free medium_ − concentration of glucose_drug-treatment_.

### Nuclear and cytoplasmic fractionation

Nuclear and cytoplasmic extraction was performed using NE-PER® Nuclear and Cytoplasmic Extraction Reagents (ThermoFisher Scientific), following the manufacturer’s instructions.

### Western blot, surface sensing of translation (SUnSET) method, co-immunoprecipitation

Cells were lysed in RIPA buffer containing protease inhibitor cocktail. Protein concentrations were determined by Bradford protein assay (Bio-Rad Laboratories, Hercules, CA). Samples were separated by SDS-polyacrylamide gel electrophoresis and transferred onto PVDF membranes (Sigma/Millipore, St. Louis, MO). Membranes were blocked with BlockPRO™ 1 min Protein-Free Blocking Buffer (Visual Protein), incubated with primary antibody (Supplementary Table 1) at 4 °C overnight, washed three times with 1× TBS containing 0.1% Tween-20, and probed with horseradish peroxide-conjugated secondary antibody (Cell Signalling Technology, Beverly, MA). Blots were developed using Enhanced Chemiluminescence reagent (Millipore).

SUnSET method measures global protein synthesis by labelling nascent polypeptides with puromycin. Kasumi‐1 cells were treated with different cabozantinib concentrations for 24 h, followed by incubation with 200 nM puromycin for 1 h. Then, cells were lysed and subjected to western blotting as described above; membranes were probed using an anti-puromycin antibody.

For co-immunoprecipitation, puromycin-labelled nascent polypeptides were pulled down using 4 μg of mouse anti-puromycin antibody; anti-AML1 antibody was used for detecting AML1-ETO in subsequent immunoblot analysis.

### Quantitative reverse transcription PCR (RT-qPCR)

After indicated treatment steps, total RNA was isolated using Nucleospin RNA kit (Macherey-Nagel, Düren, Germany) following the manufacturer’s protocol. RNA was reverse transcribed to cDNA, which was used as a template for SYBR-green-based qPCR amplification of genes of interest as previously described [12]. Primer specificity was confirmed by melting curves obtained following the reaction (for primer sequences, see Supplementary Table 2).

### RNA-sequencing analysis

Total RNA was extracted using Nucleospin RNA kit (Macherey-Nagel) after 4-h and 24-h treatments with DMSO or 100 nM cabozantinib. Sequencing was performed using an Illumina NextSeq 500 sequencer. Fastqc files of RNA-seq were sent to FastQC for a quality check; Trimmomatic was used for adapter and low-quality bases trimming. Sequencing alignment and gene expression counting was conducted using R package Rsubread with reference genome GRC37.13 from Genecode (https://www.gencodegenes.org/). Differentially expressed genes (DEGs) between DMSO and cabozantinib were identified by EBseq. A threshold of fold change ≥2 or ≤0.5 and *p* ≤ 0.05 was used to select the DEGs. Transcriptome files are provided in the GEO database (ID: GSE153853). Pathway enrichment was analysed by Metascape or gene set enrichment analysis (GSEA) using the Hallmark collections of the GSEA MSigDB 7.0.

### Immunofluorescence assay

After 4 h drug treatment, cells were harvested by Cytospin (800 × *g*, 5 min), fixed with 4% paraformaldehyde, and blocked in blocking buffer (PBS containing 0.3% Triton X-100, 0.3 M glycine, and 5% FBS) for 1 h. Then, cells were washed three times with 1× PBS containing 0.1% Triton X-100 (PBST) and incubated with anti-FOXO3a antibody at 4 °C overnight. Cells were washed again and incubated with Alexa-Fluor-labelled, goat anti-rabbit IgG secondary antibody for 1 h in the dark at room temperature. Cells were rinsed in 1× PBST and mounted on slides in EverBrite™ Hardset Mounting Medium containing DAPI for analysis using Laser TIRF (Total Internal Reflection Fluorescence) confocal microscope with 63x magnification objective.

### Flow cytometry

For cell-cycle analysis, 6-well plates were seeded with 5 × 10^5^ cells/well and incubated for 16 h before initiating the 24-h cabozantinib treatment. Then, cells were harvested, fixed in 70% ethanol for at least 16 h, and resuspended in 495 μl PBS and 5 μl RNase A (1 mg/ml) at 37 °C. Subsequent staining with DNA-binding propidium iodide (PI) was visualised by measuring fluorescence using CYTOFLEX^TM^ Flow Cytometer (Beckman Coulter Inc., Brea, CA). The cell-cycle distribution was analysed using CytExpert software.

Cell apoptosis was assessed using Fluorescein Isothiocyanate (FITC) Annexin-V Apoptosis Detection Kit I (BD Biosciences). After 72-h cabozantinib treatment, cells were washed with cold PBS and resuspended in 1× binding buffer containing Annexin-V-FITC and PI. Cell suspensions were incubated in the dark for 15 min at room temperature and measured with CYTOFLEX^TM^ Flow Cytometer. Apoptotic analysis using CytExpert software differentiates between viable (AV−/PI−), early (AV+/PI−), and late (AV+/PI+) apoptotic cells.

### In vivo efficacy of cabozantinib in murine model

Kasumi-1 cells (2 × 10^7^) were inoculated subcutaneously into the right flank of CAnN.Cg-*Foxn1*^*nu*^/CrlNarl mice (purchased from the National Laboratory of Animal Breeding and Research Centre, Taipei, Taiwan). When tumour size reached 100–200 mm^3^, mice were randomly divided into two groups receiving oral cabozantinib (10 mg/kg cabozantinib, *n* = 14; 30 mg/kg cabozantinib, *n* = 15) and one vehicle group (*n* = 12). Treatments were administered on 6 days, followed by a 1-day rest for a total course of three cycles. All mice were regularly monitored for body weight and tumour growth. Tumour volumes were calculated with the following formula: (length × width^2^) × 0.5 in millimetres. Tumour samples were fixed in formalin for histological examination. Mice were euthanised when tumour volume nearly reached 2000 mm^3^. Tissue samples were fixed in formaldehyde for H&E staining, as reported previously [15]. The xenograft experimental protocol was approved by the Institutional Animal Care and Use Committee of the College of Medicine, National Taiwan University, and conformed to the criteria outlined in the Guide for the Care and Use of Laboratory Animals prepared by the National Academy of Sciences and published by the National Institutes of Health.

### Statistical analysis

All quantitative data are expressed as means ± SDs. Differences between the treatment groups and DMSO controls were compared by Student’s *t*-test, and differences with *p* < 0.05 were considered statistically significant. Comparisons of survival curves estimated by Kaplan–Meier plots using GraphPad Prism 8.0.2 were analysed by log-rank test.

## Results

### Cabozantinib inhibited growth and triggered apoptosis of Kasumi-1 cells

**Fig. 1:**
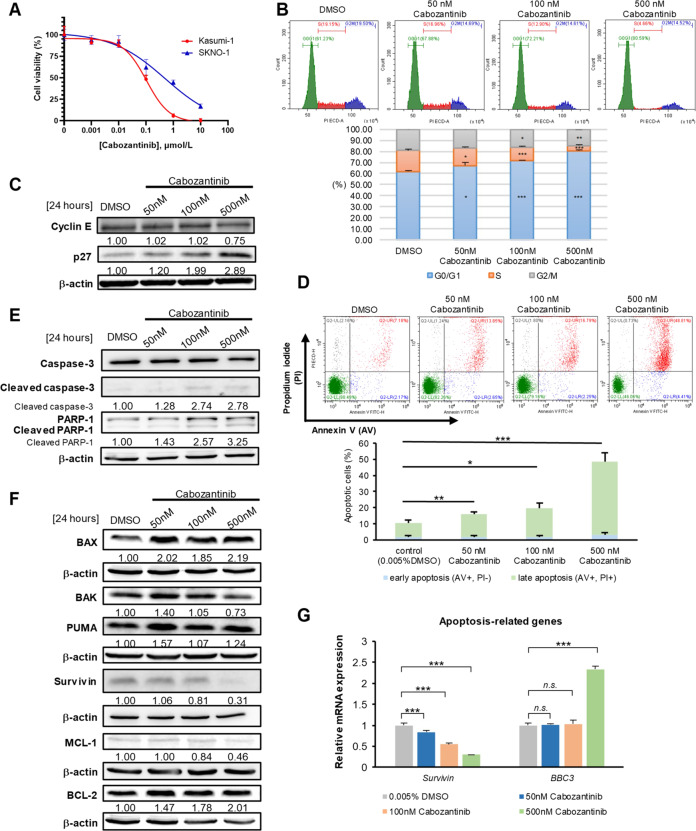
Cabozantinib induces G0/G1 cell-cycle arrest and apoptosis in KIT-mutated t(8;21) AML cells. **A** Kasumi-1 and SKNO-1 cells were treated with 0.01% DMSO (control) or various concentrations of cabozantinib for 72 h. MTS assay was performed to obtain the IC_50_ values of cabozantinib for both cell lines. The IC_50_ values were calculated using the CalcuSyn software. **B** Flow cytometric analysis after 24-h cabozantinib represents the proportion of cells at different cell-cycle stages. Histograms are one of the representative results. The data represented the averages ± SD of three independent experiments (*n* = 3). ***p < 0.001, **p < 0.01, *p < 0.05, compared with DMSO control. **C** Two G1/S transition regulators, p27 and cyclin E, were measured by western blot analysis. β-actin was loaded as a control. Signal intensity was quantified with ImageJ and normalised to β-actin and DMSO controls. **D** Flow cytometric analysis after 72-h cabozantinib treatment is presented as dot plots and the bar graphs show the proportion of cells at different apoptotic status. Each column represented the averages ± SD of three independent experiments (*n* = 3). ***p < 0.001, **p < 0.01, *p < 0.05, compared with DMSO control. **E** Western blot analysis of PARP cleavage, caspase-3 activation and **F** apoptosis-related proteins, including BAX, BAK, PUMA, Survivin, MCL-1, and BCL-2, in Kasumi-1 cells following 24-h cabozantinib treatment. **G** *BBC3* and *survivin* relative mRNA expression were analysed by RT-qPCR analysis following 24-h cabozantinib treatment. Each column represented the averages ± SD of three independent experiments (*n* = 3). ***p < 0.001, **p < 0.01, *p < 0.05, compared with DMSO control.

Significant cytotoxicity of cabozantinib was detected in Kasumi-1 and SKNO-1 with IC_50_ values of 88.06 and 503.55 nM, respectively (Fig. 1A), which were within clinically achievable plasma levels [16, 17]. Evaluation of the cell proliferation kinetics with the trypan blue exclusion assay revealed that cabozantinib inhibited the proliferation of Kasumi-1 cells in a dose- and time-dependent manner. The doubling time of 36.95 h in DMSO was increased to 51.52 and 83.66 h in 50 and 100 nM cabozantinib, respectively (Supplementary Fig. 2A); growth inhibition and prolonged doubling time were also observed in cabozantinib-treated SKNO-1 cells (Supplementary Fig. 2B). To elucidate the molecular mechanism of the antiproliferative effects of cabozantinib, cell cycle and apoptosis analyses were performed. We found that treatment of Kasumi-1 cells with 50 nM cabozantinib induced significant G_0_/G_1_ cell-cycle arrest within 24 h in a dose-dependent manner (Fig. 1B). Cabozantinib downregulated the G1/S transition regulator cyclin E and upregulated the cyclin-dependent kinase (CDK) inhibitor p27 (Fig. 1C), which led to G_0_/G_1_ cell-cycle arrest, suggesting that their dysregulation contributed to the inhibitory effects.

The apoptosis analysis detected dose-dependently increased percentages of annexin-V and PI double-positive Kasumi-1 cells following a 72-h cabozantinib treatment (Fig. 1D). Immunoblotting analysis indicated that cabozantinib treatment increased cleavage of caspase-3 and PARP-1 (Fig. 1E), implying the treatment induced the caspase-3-dependent proteolytic cascade. Further analysis of apoptosis-associated proteins revealed that cabozantinib treatment upregulated pro-apoptotic proteins BAX and PUMA but hardly affected BAK (Fig. 1F). Moreover, cabozantinib treatment downregulated anti-apoptotic proteins survivin and MCL-1, whereas BCL-2 remained unchanged. The treatment also significantly inhibited *survivin* mRNA expression and induced *PUMA* (*BBC3*) mRNA expression (Fig. 1G), denoting that *BBC3* and *survivin* might be under transcriptional control. These findings suggested that the increase in cleaved caspase-3 and cleaved PARP-1, as well as in apoptosis-related proteins, could contribute to mediating apoptosis in cabozantinib-treated Kasumi-1 cells.

### Cabozantinib exerted inhibitory activity against KIT and its downstream signals

**Fig. 2:**
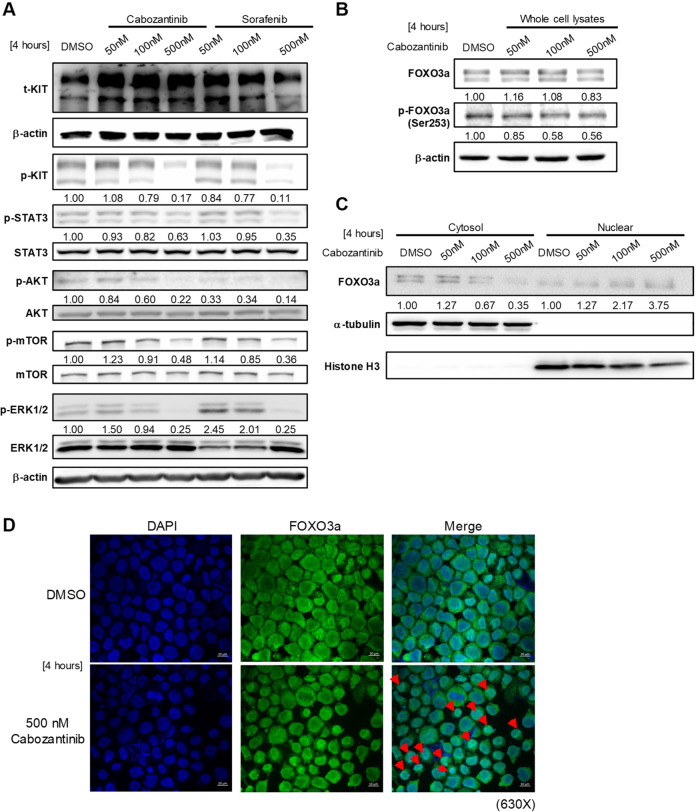
Cabozantinib inhibits the phosphorylation of KIT-mediated signalling molecules and elicits FOXO3a nuclear localisation in Kasumi-1 cells. Cells were treated with different concentrations of cabozantinib and sorafenib for 4 h. Sorafenib, a known a KIT inhibitor, was used as a positive control. **A** Phosphorylation of KIT and its downstream STAT3, AKT/mTOR and ERK molecules in Kasumi-1 cells were measured by western blots. Kasumi-1 cells were treated with DMSO or various concentrations of cabozantinib for 4 h. **B** Western blots of whole-cell lysates and **C** subcellular fractions of Kasumi-1 cells. Histone H3 (nuclear) and α-tubulin (cytosol) served as loading controls for fractionation. Signal intensity was quantified with ImageJ and normalised to the loading control and control group. **D** Confocal microscopy images of DMSO-treated or cabozantinib-treated Kasumi-1 cells show signals for DAPI stained nuclei (blue) and FOXO3a (green), and the merged images (overlay). The red arrowheads point to representative cells showing increased nuclear FOXO3a levels. Scale bars, 10 μm.

Because of the constitutive ligand-independent activation of KIT-induced downstream oncogenic signalling pathways, including the STAT3, PI3K/AKT, and RAS/RAF/MEK/ERK pathways [18], we initially examined the cabozantinib-induced inhibition of the phosphorylation of KIT and downstream signalling pathways. A 4-h cabozantinib treatment decreased phosphorylation of KIT and its downstream signalling pathways, including PI3K/AKT/mTOR, ERK1/2, and STAT3, in Kasumi-1 cells (Fig. 2A). However, the inhibitor’s effect on SKNO-1 cells was limited to a decreased KIT phosphorylation (Supplementary Fig. 3A), indicating that SKNO-1 cells might be less sensitive to cabozantinib than Kasumi-1 cells.

Forkhead box O3a (FOXO3a), a member of the FOXO subfamily of forkhead transcription factors, is a canonical downstream target of the PI3K/AKT pathway involved in the cell cycle [19–21]. AKT phosphorylates FOXO3a at serine-253, disrupting its rate of nuclear export [22]. To elucidate whether the effect of cabozantinib on Kasumi-1 cells involved FOXO3a, an analysis of whole extracts of DMSO- or cabozantinib-treated Kasumi-1 cells revealed that the latter had lower levels of phosphorylated FOXO3a than the former (Fig. 2B). We further assessed the subcellular distribution of FOXO3a by examining cytoplasmic and nuclear extracts of pre-treated and treated Kasumi-1 cells. Western blotting revealed that cabozantinib treatment diminished the FOXO3a level in the cytoplasmic fraction, while the FOXO3a level in the nuclear fraction was dose-dependently increased (Fig. 2C). Additionally, confocal immunofluorescence analysis also revealed that endogenous FOXO3a resided almost exclusively in the cytoplasm of untreated cells, whereas nuclear staining was negligible. The subcellular distribution of FOXO3a became increasingly localised to the nucleus after cabozantinib treatment (Fig. 2D), although some punctate perinuclear FOXO3a staining remained apparent; indicating FOXO3a was redistributed from cytoplasm to nucleus. Nuclear FOXO3a localisation can promote CDK inhibitor p27 expression, preventing the activation of G1/S regulator cyclin E-CDK2 complexes [23], which induced cell-cycle arrest. We concluded that cabozantinib could translocate FOXO3a protein from the cytoplasm to the nucleus by inhibiting PI3K/AKT signalling, promoting p27 expression, and inhibiting cyclin E (Fig. 1C), which in turn induced cell-cycle arrest.

### Cabozantinib downregulated AML1-ETO protein expression by interfering with oncofusion protein synthesis

**Fig. 3:**
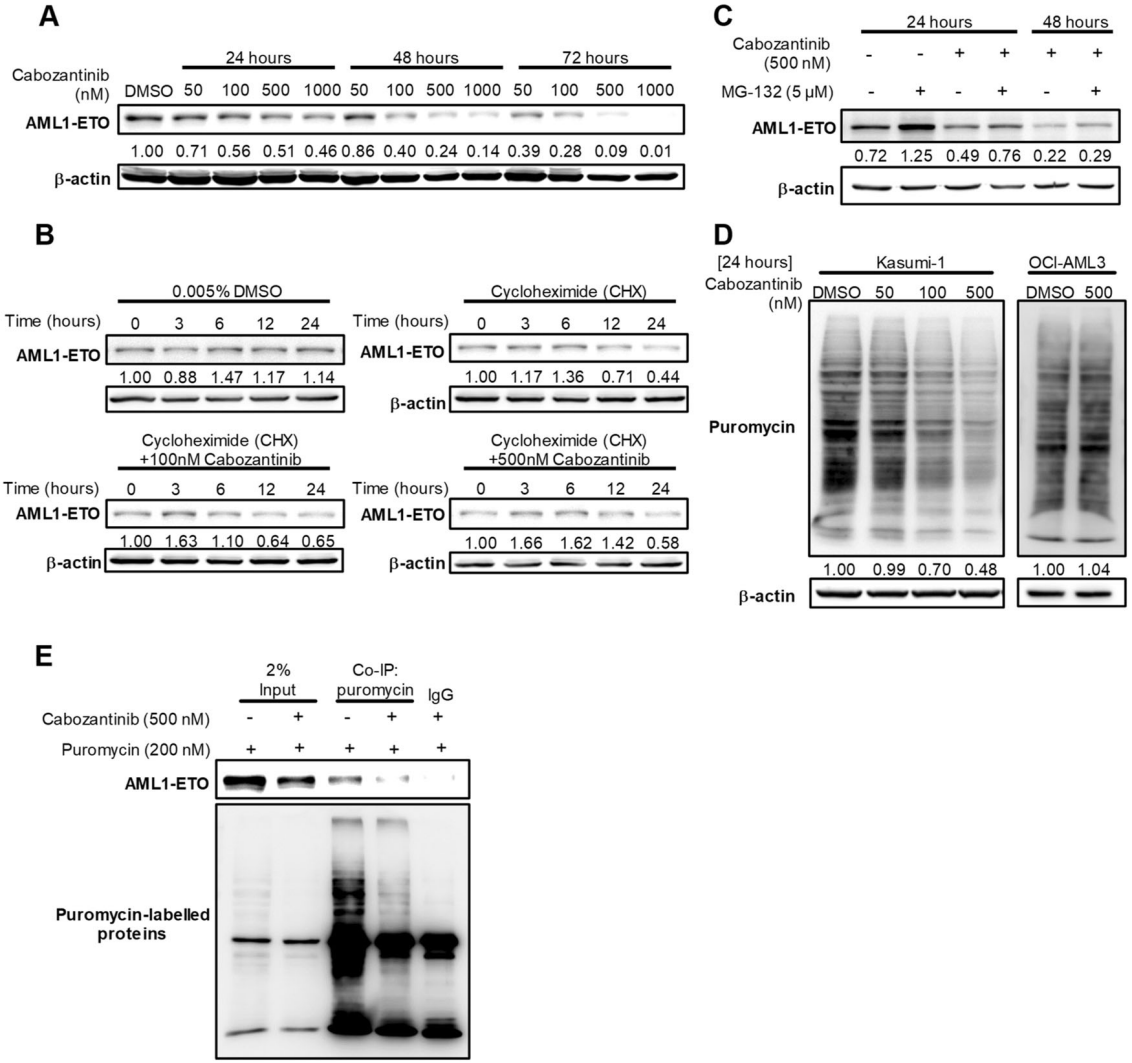
Molecular mechanisms of cabozantinib-induced AML1-ETO downregulation. **A** Kasumi-1 cells were treated with DMSO or various concentrations of cabozantinib for 24 h, 48 h, and 72 h before harvesting. Western blot analysis of the time course of changes in protein levels of AML1-ETO. **B** Western blot analysis of the time course of changes in the protein level of AML1-ETO after DMSO (control), CHX alone or in combination with cabozantinib. Signal intensity was quantified with ImageJ and normalised to β-actin and control (0-h) group. **C** The protein expression of AML1-ETO was measured in Kasumi-1 cells, which were treated with 500 nM cabozantinib for indicated hours before harvested for western blot analysis. The proteasome inhibitor MG-132 (5 μM) was added 4 h before cell harvest. **D** Monitoring protein synthesis in Kasumi-1 and OCI-AML3 cells by puromycin labelling. β-actin was loaded as a control. Signal intensity was quantified with ImageJ and normalised to β-actin and DMSO controls. **E** Co-immunoprecipitation (co-IP) followed puromycin-labelled de novo synthesis protein showed decreased AML1-ETO protein expression level. Mouse IgG was used as a negative control of co-IP.

AML1-ETO is an oncofusion protein critical for leukaemogenesis through impeding normal AML1 function [24]. We observed dose- and time-dependent dysregulation of AML1-ETO in cabozantinib-treated Kasumi-1 cells (Fig. 3A). To elucidate the molecular mechanisms of cabozantinib-induced AML1-ETO downregulation, *AML1-ETO* mRNA expression in cabozantinib-treated cells was quantified by RT-qPCR. Interestingly, cabozantinib did not affect the *AML1-ETO* expression at mRNA levels (Supplementary Fig. 4A), suggesting a transcription-independent regulation.

Protein abundance mirrors the integration of protein degradation and protein synthesis rates [25]. We disrupted de novo protein synthesis with CHX and found that the AML1-ETO protein half-life was barely altered after cabozantinib treatment (Fig. 3B), excluding the possibility that cabozantinib-associated AML1-ETO reduction was due to an accelerated turnover. MG-132, a proteasome inhibitor, was used to block proteasome-dependent degradation. AML1-ETO accumulation was less reduced by MG-132/cabozantinib co-treatment than by MG-132 alone, suggesting a potential role of cabozantinib in hindering protein synthesis (Fig. 3C). Protein synthesis quantification using SUnSET assay [26]. revealed that cabozantinib dose-dependently reduced global protein synthesis in Kasumi-1 cells but not in cabozantinib-nonresponsive OCI-AML3 (KIT wild type) cells (Fig. 3D). Further immunoprecipitation followed by puromycin-labelling method directly assessed AML1-ETO synthesis, demonstrating that cabozantinib reduced the AML1-ETO protein synthesis (Fig. 3E). It is known that caspase-3 and heat shock protein 90 (HSP90) can trigger AML-ETO oncoprotein degradation [27, 28]. Our results demonstrated that Z-VAD-FMK, a pan-caspase inhibitor, inhibited cabozantinib-induced caspase-3 cleavage but could not rescue cabozantinib-induced AML1-ETO downregulation in Kasumi-1 cells (Supplementary Fig. 4B). Furthermore, cabozantinib failed to affect HSP90 protein expression (Supplementary Fig. 4C). Thus, cabozantinib treatment downregulated AML1-ETO protein expression by impairing oncofusion protein synthesis rather than enhancing proteasome-dependent degradation.

### Transcriptomic analyses indicated that cabozantinib could repress glycolytic features in Kasumi-1 cells

**Fig. 4:**
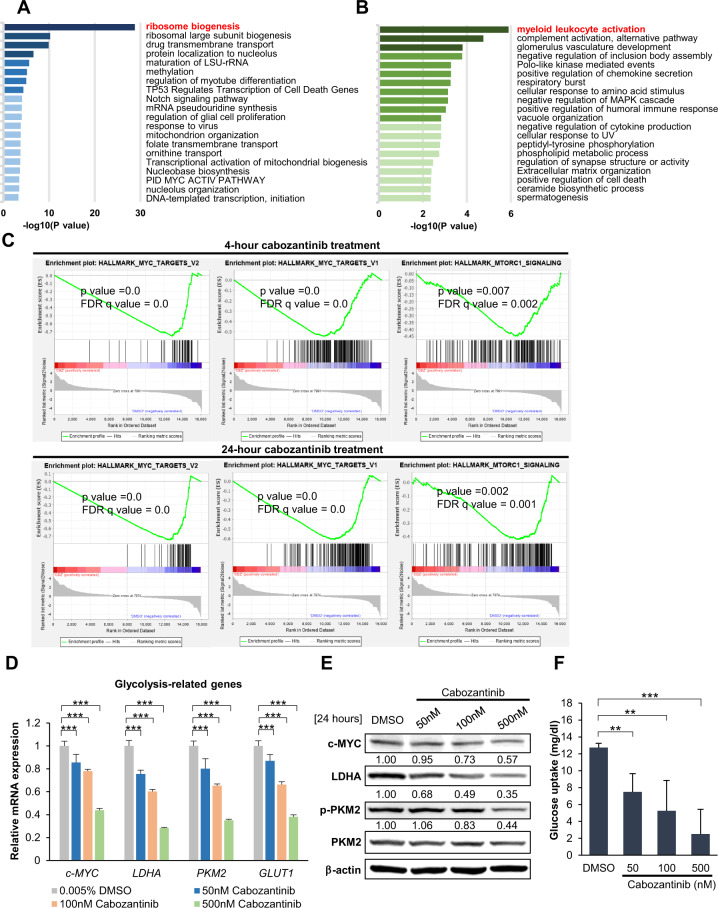
Genome-wide assessment of the anti-leukaemia effects. RNA-seq was performed on Kasumi-1 cells that were either untreated or treated with cabozantinib (4 and 24 h at 100 nM). **A** Gene ontology (GO) analysis using Metascape was performed to analyse genes markedly enriched compared with the DMSO group, after the 4-h cabozantinib treatment and **B** the 24-h cabozantinib treatment. **C** Gene set enrichment analysis (GSEA) was also performed to interpret gene expression data (4-h cabozantinib-treated, 24-h cabozantinib-treated). The common downregulated pathways of the two groups were identified by GSEA analysis using the Hallmark collections of the GSEA MSigDB 7.0. In addition, Kasumi-1 cells were treated with DMSO (control) or various concentrations of cabozantinib for 24 h, and harvested for western blot analysis, RT-qPCR, and glucose uptake assay. **D** RT-qPCR analysis of glycolysis-related genes. Transcript levels were normalised to those of actin, and the relative mRNA expression was calculated using the 2^−^^ΔΔCT^ method. Each column represented the averages ± SD of three independent experiments (*n* = 3). ***p < 0.001, **p < 0.01, *p < 0.05, compared with DMSO control. **E** The cell lysates mentioned above were subjected to immunoblotting with c-MYC, LDHA, p-PKM2, PKM2, and β-actin antibodies. β-actin was loaded as control. Signal intensity was quantified with ImageJ and normalised to β-actin and DMSO controls. **F** Determination of glucose uptake by cabozantinib-treated Kasumi-1 cells. Values represent mean ± SD. ***p < 0.001, **p < 0.01, *p < 0.05, compared with DMSO control.

We searched for global transcriptomic changes in cabozantinib-treated Kasumi-1 cells to identify any additional mechanism induced by cabozantinib treatment. We conducted an RNA-seq analysis of cells treated with 100 nM cabozantinib or DMSO for 4 and 24 h, representing short-term and long-term responses, respectively. We identified 538 and 253 DEGs between cabozantinib-treated and control cells after the 4 and 24-h treatment, respectively. By using the gene ontology (GO) approach to identify enriched pathways in Metascape, we found that the top-ranked enriched pathways after the 4 and 24-h cabozantinib treatment were related to ribosome biogenesis (Fig. 4A) and myeloid leukocyte activation, respectively (Fig. 4B).

Besides Metascape, GSEA was applied as another pathway enrichment analysis tool for transcriptomic data interpretation. Intriguingly, functional analysis of Kasumi-1 cells after the 4 and 24-h cabozantinib treatment identified three significantly downregulated gene sets—MYC_targets_V2, MYC_targets_V1, and MTORC1 signalling. These three gene sets, which are strongly linked to cell metabolism [29–32], were similarly enriched regardless of the treatment period (Fig. 4C), suggesting that cabozantinib affected metabolic activity. Subsequently, we examined glycolysis-related genes and protein expression levels after 24-h cabozantinib treatment by RT-qPCR and western blotting, respectively. Interestingly, glycolysis-related genes, including *PKM2*, *LDHA*, and *GLUT1*, were suppressed by cabozantinib exposure (Fig. 4D), and the protein expression profiles were similar (Fig. 4E). Consistent with reduced *GLUT1* mRNA expression induced by cabozantinib, a tendency to less glucose uptake activity following cabozantinib treatment was observed (Fig. 4F). Consequently, we suggest that cabozantinib triggered p-mTOR inhibition following KIT suppression, which then abrogated downstream glycolysis target genes and deprived leukaemic cells of intracellular glucose transport necessary for cell survival and proliferation.

### Cabozantinib inhibited AML1-ETO-mediated transcriptome

**Fig. 5:**
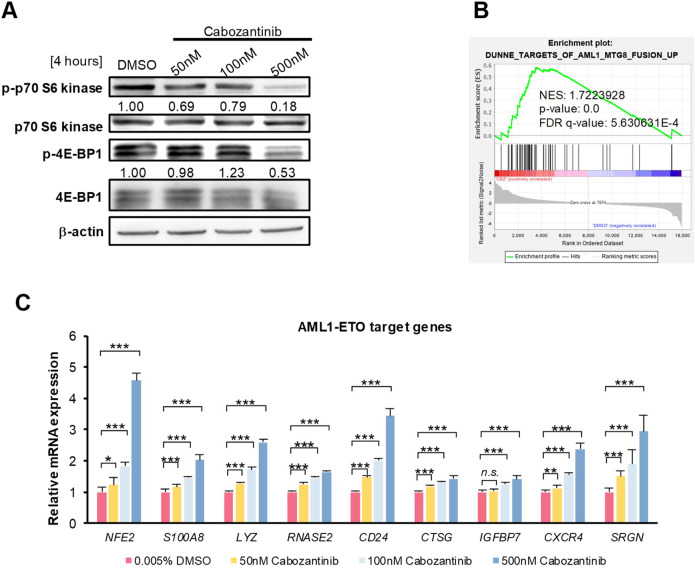
Cabozantinib inhibits AML1-ETO-mediated transcriptome. **A** Western blot analysis of the phosphorylation of S6K and 4E-BP1. β-actin was loaded as a control. Signal intensity was quantified with ImageJ and normalised to β-actin and DMSO controls. **B** GSEA analysis identified genes upregulated following cabozantinib treatment were similar to a pre-defined gene set (DUNNE_TARGETS_OF_AML1_MTG8_FUSION_UP) comprising genes upregulated in Kasumi-1 cells after AML1-ETO knockdown by siRNA. **C** Relative mRNA expression of AML1-ETO-suppressed genes related to cell differentiation was analysed following 24-h of cabozantinib treatment in Kasumi-1 cells. Actin served as the internal control. Transcript levels were normalised to those of *actin* and the relative mRNA expression was calculated using the 2^−ΔΔCT^ method. Each column represented the averages SD of three independent experiments (*n* = 3). ***p < 0.001, **p < 0.01, *p < 0.05, compared with DMSO control.

Our Metascape analyses unravelled the ribosome biogenesis inhibition after cabozantinib treatment (Fig. 4A). Generally, uncontrolled cellular proliferation is a hallmark of cancer, maintained through high levels of protein synthesis and ribosome biogenesis. Therefore, we investigated two key modulators of ribosome biogenesis, the RP S6 kinases (S6K1/2) and the protein initiation factor 4E binding proteins (4E-BP1/2/3). Immunoblotting showed that 4-h cabozantinib treatment decreased S6K and 4E-BP1 phosphorylation (Fig. 5A), consistent with previous results about disrupting oncofusion protein synthesis (Fig. 3E).

Additionally, GSEA against a ranked list of known AML1-ETO-targeted genes generated from siRNA-mediated *AML1-ETO* knockdown in Kasumi-1 cells [33] was compatible with our transcriptomic results obtained from 24-h cabozantinib-treated Kasumi-1 cells (Fig. 5B), confirming that cabozantinib treatment resembled the effect of *AML1-ETO* knockdown. Furthermore, several myeloid differentiation-related genes originally suppressed by *AML1-ETO* were dose-dependently upregulated after a 24-h cabozantinib treatment (Fig. 5C), consistent with the transcriptomic results (Fig. 4B). In summary, cabozantinib not only diminished AML1-ETO protein synthesis, possibly through the mTOR/S6K/4E-BP1 axis, but also upregulated myeloid differentiation-related gene expression.

### Cabozantinib suppressed the growth of subcutaneous Kasumi-1 xenograft tumours

**Fig. 6:**
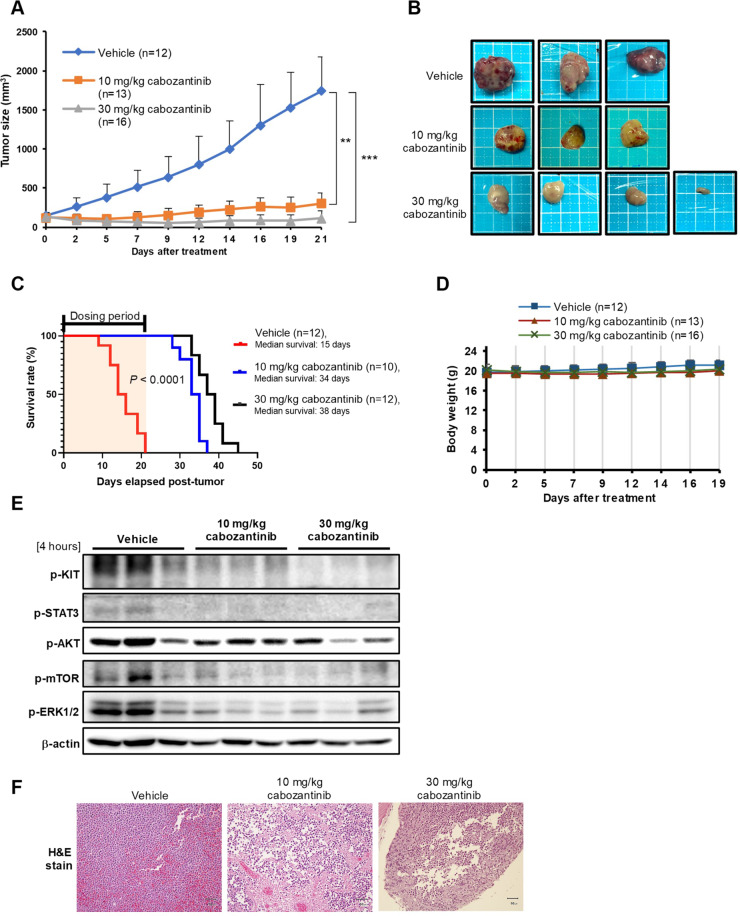
Effects of cabozantinib in a Kasumi-1 xenograft mouse model. **A** Cabozantinib causes prominent regression of subcutaneous Kasumi-1 tumours in athymic nude mice. The results were plotted as mean tumour size ± SD of three groups over time. **B** The tumours were dissected and photographed at 4 h following the last oral administration with cabozantinib. **C** When the tumour volume reached 1000 mm^3^ the mice were considered death. Comparisons of survival curves estimated by Kaplan–Meier plots. **D** No obvious body weight loss was observed during treatments. **E** Tumours were recovered from Kasumi-1-bearing mice after 4 h of treatment with vehicle or cabozantinib (*n* = 3/group); then tumour lysates were extracted to analyse phosphorylated KIT, STAT3, AKT/mTOR and ERK molecules by western blot. **F** H&E staining was performed on Kasumi-1 tumours treated with cabozantinib or vehicle. Scale bars, 50 μm.

We established Kasumi-1 xenografts in nude mice to assess the potential in vivo antitumor efficacy of cabozantinib. In agreement with in vitro results, once-daily gavage of 10 or 30 mg/kg of cabozantinib apparently retarded tumour growth and prolonged survival (Fig. 6A–C) without obvious changes in body weight throughout the treatment course (Fig. 6D). Western blot analysis of the expression of KIT-mediated signalling pathways in tumour tissues demonstrated that cabozantinib treatment also decreased p-KIT, p-STAT3, p-AKT, p-mTOR, and p-ERK1/2 expression levels in vivo (Fig. 6E). After the three-cycle cabozantinib treatment, tumour cells were shrunk and loosely arranged, compared with the observations in vehicle-treated mice (Fig. 6F).

## Discussion

In AML, *KIT* mutation is the most common cooperating mutation in AML1-ETO leukaemia, adversely affecting the disease outcome [3, 34]. Due to the high incidence of activating *KIT* mutations in AML1-ETO leukaemia, KIT signalling is considered a superb target. Presently, clinical trials of dasatinib, a known KIT inhibitor in AML, are being conducted [35]; however, the underlying molecular mechanisms employed by KIT inhibitors in AML remain obscure. Over the past decades, TKIs have been developed as critical cancer therapy components based on the discovery that most disease situations emanate from signal transduction pathways gone awry. To date, more than 130 emerging TKIs exhibiting great efficacy are approved for clinical trials [16, 36], of which are KIT-targeted drugs [37]. In the present study, we initially elucidated that cabozantinib inhibited KIT phosphorylation and its downstream signalling, including PI3K/AKT/mTOR, as well as suppressed ribosome biogenesis and AML1-ETO protein synthesis, which subsequently increased the expression of AML1-ETO target genes.

Our global transcriptomic analysis based on Metascape enrichment analyses found that the top pathway associated with those downregulated DEGs was ribosome biogenesis. The ribosome is a complex molecular machine responsible for protein synthesis in every living cell; additionally, ribosome biogenesis is the machinery that performs protein synthesis. Here we found that cabozantinib inhibited phosphorylation of p70S6K and 4E-BP1, and reduced the protein synthesis of AML1-ETO without increasing its degradation, unlike several anticancer agents that facilitate proteasomal degradation of AML1-ETO oncoprotein [38–40]. In addition, we clearly excluded the possibility that AML1-ETO dysregulation could be attributed to the cabozantinib-mediated acceleration of the turnover rate by using protein synthesis inhibitor CHX. We further proved that cabozantinib caused AML1-ETO reduction through interference with oncofusion protein synthesis based on experiments using proteasome inhibitor MG-132 and the SUnSET method. A similar drug effect on fusion protein synthesis was also noted in chronic myeloid leukaemia [41]. Specifically, homoharringtonine, a cephalotaxine ester derived from the evergreen tree *Cephalotaxus harringtonia* [42], inhibited protein synthesis and reduced the BCR-ABL protein level. Here, we inferred that cabozantinib interfered with AML1-ETO protein synthesis, likely by blocking the KIT/mTOR/S6K/4E-BP1 pathway.

In association with the KIT mutation, a study on transformed isogenic BaF3 cells whose proliferation depends on the wild type or oncogenic KIT mutants revealed that cabozantinib was effective against the N822K activation mutants which were resistant to imatinib and sunitinib in vitro and in vivo, demonstrating the efficacy of cabozantinib to cells carrying only KIT N822K [43]. As stated above, cabozantinib abolished KIT-mediated signalling, hampered ribosome biogenesis and AML1-ETO protein synthesis, and increased the expression of AML1-ETO target genes. We thus concluded that cabozantinib targeted KIT mutants and inhibited ribosome biogenesis directly or indirectly, along with AML1-ETO protein synthesis, and eventually reactivated the expression of those differentiation-related genes suppressed by AML1-ETO.

Our study, including deep transcriptome analysis via RNA-seq to provide unparalleled insights, along with the opportunity to assess the novel effect of cabozantinib, which underpinned our drug repurposing strategy. GSEA analyses identified the downregulation of mTOR- and MYC-related signalling pathways in Kasumi-1 cells after 4 or 24-h treatment with cabozantinib. Of note, these are compatible with the observations about glycolysis perturbation and AML1-ETO protein synthesis interference caused by cabozantinib.

High glycolytic metabolism, along with mTORC1 hyperactivation, is common in AML [44]. Here we specifically detected the downregulation of glucose transporter 1 (*GLUT1*), pyruvate kinase isoform 2 (*PKM2*), and lactate dehydrogenase A (*LDHA*), which were c-MYC-transcriptionally modulated downstream glycolytic genes that reportedly function as oncogenic drivers of glycolysis with augmented glucose uptake and fast glucose-to-lactate conversion [32]. GLUT1 is a cellular transmembrane glucose transporter with a relatively high glucose affinity, which is vital for tumour cells that are highly glucose-dependent for energy [45]. As a notorious hallmark of cancer, unbridled cellular proliferation is sustained by increasing the metabolism of glucose and addiction to a high level of protein synthesis, as well as ribosome biogenesis [46, 47]. mTOR and MYC are pivotal modulators in these oncogenic transformation events [48–50]. Increasing evidence suggests that oncogene-driven metabolic alterations create a distinct metabolic state that can potentially targeted by anticancer agents [51, 52]. As reported previously, compound 2-deoxy-d-glucose (2-DG), a glycolysis inhibitor [53], exhibited marked cytotoxicity in *KIT*-mutated Kasumi-1 cells compared with other leukaemic cell lines [54], implying that Kasumi-1 cells might be particularly vulnerable to glycolysis inhibition. As such, inhibitory effects of cabozantinib on glycolysis would aggregate vulnerabilities and merit further combination therapies.

In this study, cabozantinib displayed cytotoxic efficacy in two human AML cell lines, Kasumi-1 and SKNO-1, carrying the t(8;21) and *KIT* N822K mutation, but the susceptibility of the two cell lines to cabozantinib differed, with IC_50_ values of 88.06 and 503.55 nM, respectively. The factors causing substantially different responses in the two cell lines should be explored further.

In conclusion, our study not only provides a rationale for cabozantinib-based therapy but also reveals a novel mechanism by which cabozantinib exerts therapeutic efficacy in KIT-driven t(8;21) AML. Through inhibiting KIT tyrosine kinase, downstream AKT/mTOR, STAT3 and ERK were suppressed. Subsequent c-MYC downregulation was probably linked to anti-leukaemia activities, including cell-cycle arrest and glycolysis inhibition, along with ribosome biogenesis repression, which diminished AML1-ETO protein synthesis.

## Supplementary information

Supplementary Materials

Data Set 1

Data Set 2
