# Supplementary material for: Repurposing cabozantinib with therapeutic potential in KIT-driven t(8;21) acute myeloid leukaemias
Source: Cancer Gene Ther. 2021 Apr 8;29(5):519–32. doi: 10.1038/s41417-021-00329-1 (PMC9113930; doi:10.1038/s41417-021-00329-1)
Supplement: Supplementary file 1 — Supplementary Materials [file 41417_2021_329_MOESM1_ESM.docx]

**Supplementary Materials**

**Materials and methods.**

*Plotting of cabozantinib IC_50_ data*

Data was derived from Genomics of Drug Sensitivity in Cancer (GDSC, https://www.cancerrxgene.org/), and box plots as well as scatter plots were then drawn by R language and package viridis.

**Figure and Figure legends**

**Supplementary fig. 1.**

**Integrated assessment of drug response data for cabozantinib in cancers.**

**(A)** Box plots of the IC_50_ values of cabozantinib in different cell lines in GDSC database. Boxes are colored by cancer type (TCGA classification) **(B)** A scatter plot of cell line IC_50_ values for four kinds of neoplastic hematologic disorders (ALL, CLL, LAML, LCML) in GDSC database. The stable plasma concentration (~1000 nM) of cabozantinib in human body presents in gray line. **(C)** The list of LAML cell lines and their IC_50_ values of cabozantinib.

**
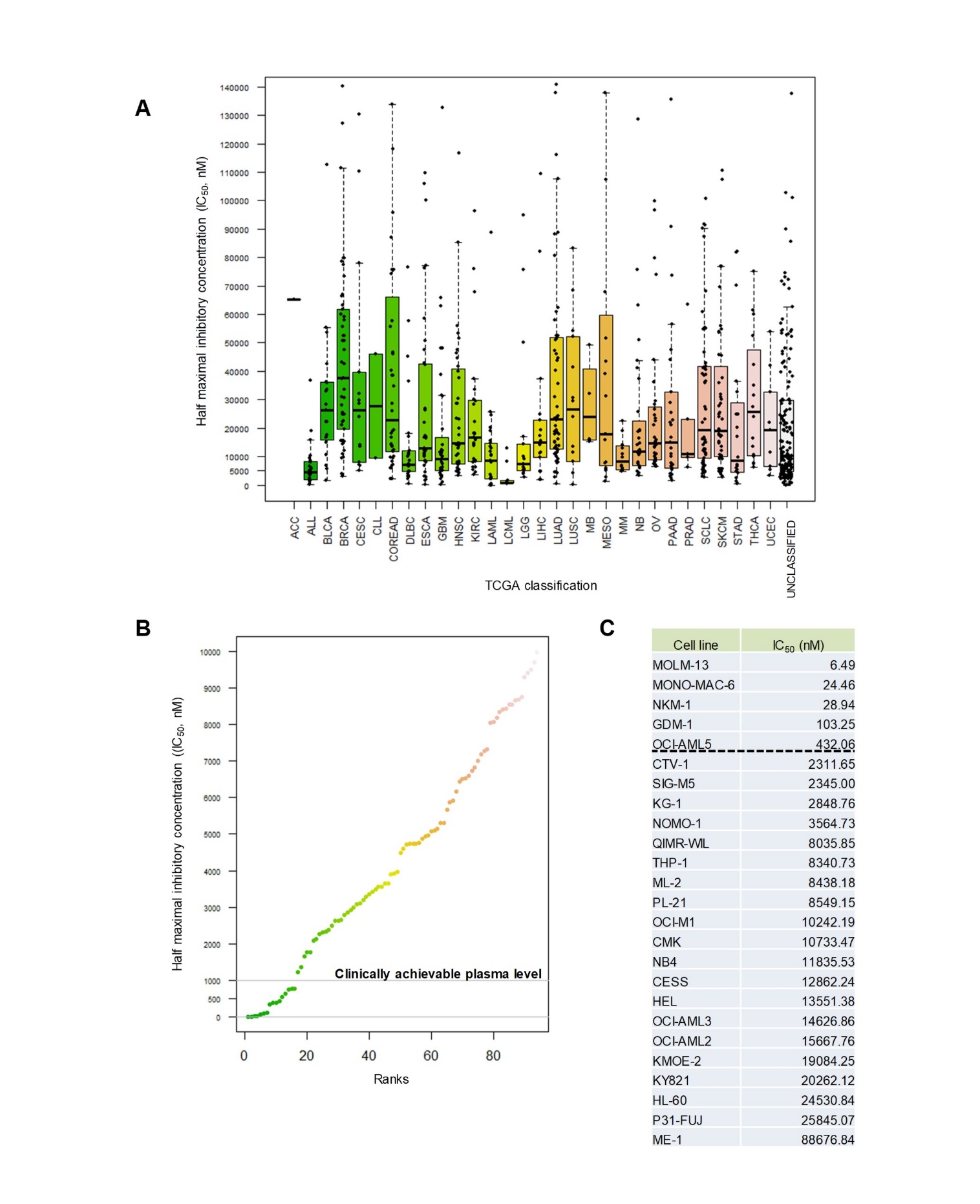
**

**Supplementary fig. 2.**

**Cabozantinib inhibits cell proliferation and prolongs doubling time in two human t(8;21) AML cell lines.**

**(A)** Kasumi-1 and **(B)** SKNO-1 cells were treated with either DMSO (control) or different concentrations of cabozantinib for 0 hour, 24 h, 48 h, 72 h and 96 h. Trypan blue exclusion assay was performed to calculate live cells in different time points. The doubling-time was calculated from the cell growth curve during 4 days. Equation for doubling time = (t-t^0^) log2/ logN – logN^0^ (t, final time; unit, hour; t^0^, initial times; N, 96-hour cell number; N^0^, 0-hour cell number). The doubling time was calculated and shown in the table below the figure. ***p < 0.001, **p < 0.01, *p < 0.05 compared to the control group.


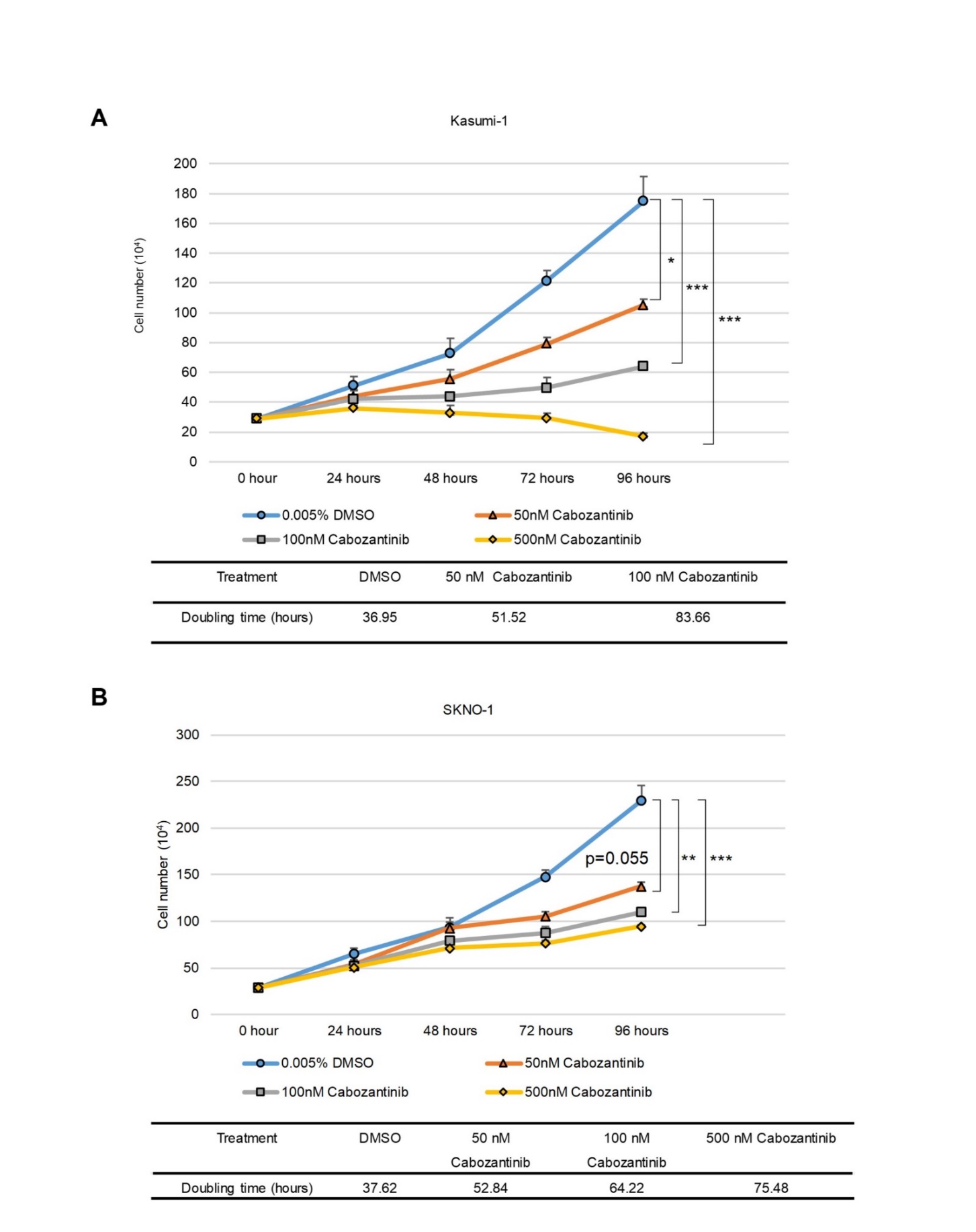


**Supplementary fig. 3.**

**Cabozantinib inhibits proliferation but only induced slightly cell apoptosis in SKNO-1 cells**

**(A)** Intracellular signaling of the KIT and downstream signaling pathways in 4-h cabozantinib-treatment SKNO-1 cells were measured by Western blots. **(B)** SKNO-1 cells were treated with either DMSO or different concentrations of cabozantinib for 24 h, then stained with PI and analysed by flow cytometry. Histograms (upper figures) show the PI fluorescence intensity; bar graphs show the proportion of cells at different cell-cycle stages. **(C)** After 72-h treatment of cabozantinib, SKNO-1 cells then stained with Annexin V and PI, and analysed by flow cytometry. Flow cytometric analyses are presented as dot plots.

**
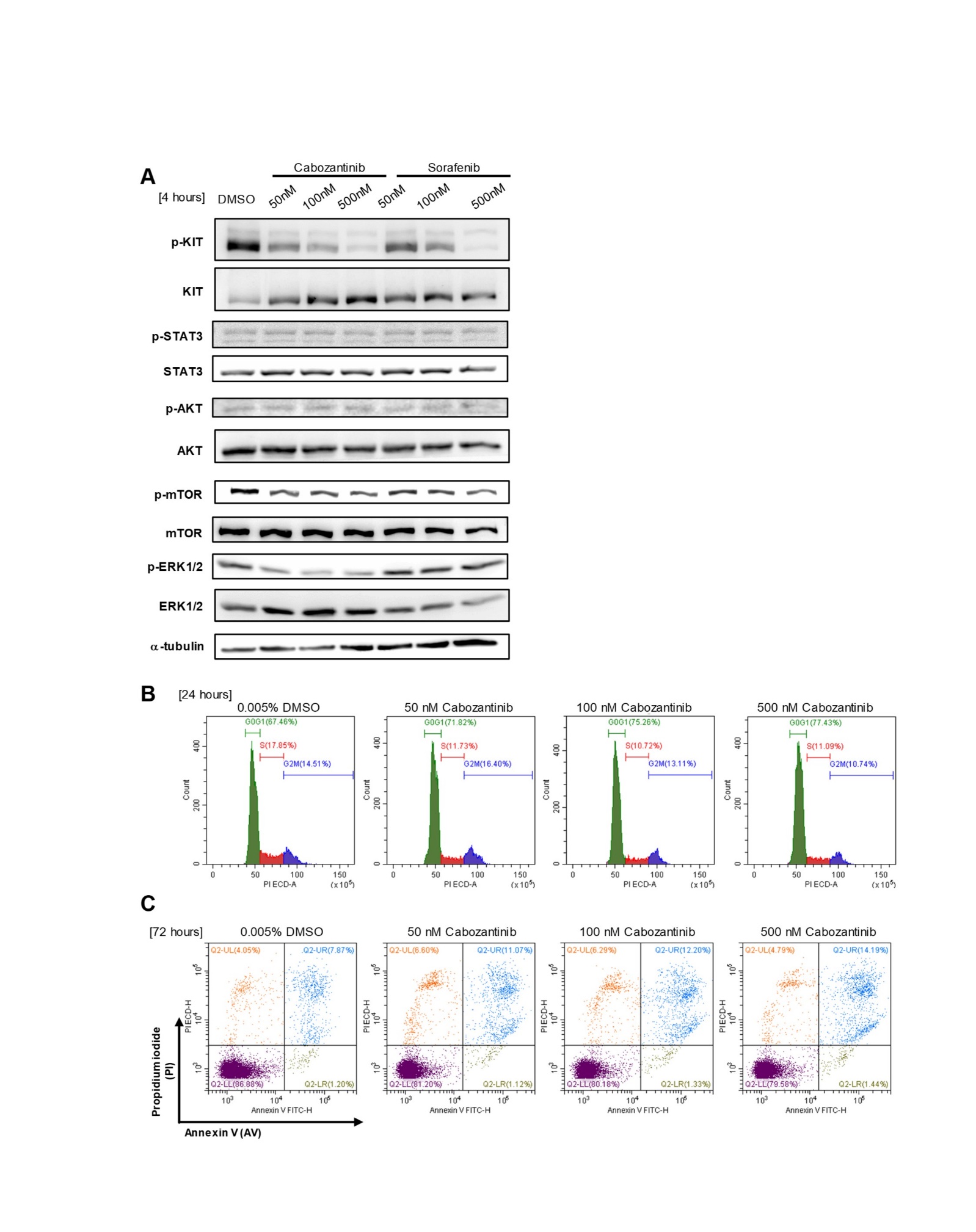
**

**Supplementary fig. 4.**

**Cabozantinib‐induced downregulation of AML1‐ETO in Kasumi-1 cells is independent of transcription regulation, activated caspase‐3 and HSP90 regulation.**

**(A)** Relative mRNA expression of *AML1-ETO* was analysed following 24-h of cabozantinib treatment in Kasumi-1 cells. *Actin* served as an internal control. Transcript levels were normalised to those of *actin* and the relative mRNA expression was calculated using the 2^-ΔΔCT^ method. Values represent mean ± SD. **(B)** Kasumi-1 cells were treated with 500 nM cabozantinib or vehicle (DMSO) for 24 hours after pretreatment with either Z-VAD-FMK or vehicle (DMSO) for 1 hour. Western blot analysis of the changes in protein levels of AML1-ETO. β-actin was loaded as a control. **(C)** Determination of the HSP90 protein expression after 24-h treatment of cabozantinib by Western blotting. β-actin was loaded as a control. Signal intensity was quantified with ImageJ and normalised to β-actin.


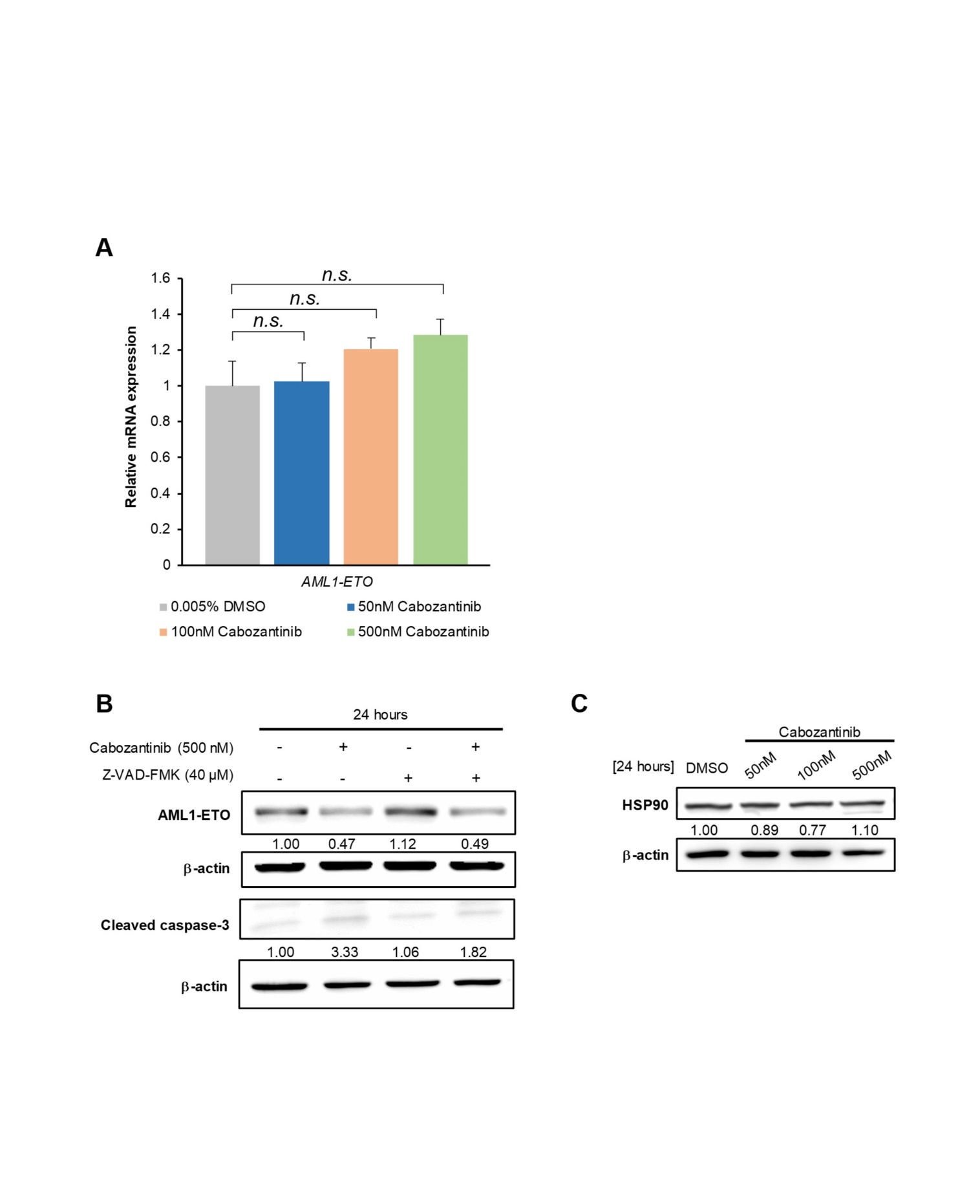


**Tables**

**Supplementary table 1.**

Primary antibodies used in this research.

| Product | Brand | Catalog no. # | Source |
| --- | --- | --- | --- |
| Phospho-c-KIT (Tyr719) | Cell signaling | 3391 | Rabbit |
| c-KIT | Cell signaling | 3308 | Mouse |
| Phospho-mTOR (Ser2448) | Cell signaling | 2971 | Rabbit |
| mTOR | Cell signaling | 2972 | Rabbit |
| Phospho-Stat3 (Tyr705) | Cell signaling | 9131 | Rabbit |
| Stat3 | Cell signaling | 12640 | Rabbit |
| Phospho-Akt (Ser473) | Cell signaling | 4058 | Rabbit |
| Akt | Cell signaling | 9272 | Rabbit |
| Phospho-p44/42 MAPK (Erk1/2) (Thr202/Tyr204) | Cell signaling | 4370 | Rabbit |
| p44/42 MAPK (Erk1/2) | Cell signaling | 4695 | Rabbit |
| FOXO3a  p-FOXO3a (Ser253) | Cell signaling  Cell signaling | 2497  9466 | Rabbit  Rabbit |
| α-tubulin | Genetex | 628802 | Mouse |
| β-actin | Genetex | 109639 | Rabbit |
| Histone H3 | Genetex | 122148 | Rabbit |
| Cyclin E1 | Cell signaling | 4129 | Mouse |
| p27 | Santa cruz | 1641 | Mouse |
| Caspase-3 | Genetex | 110543 | Rabbit |
| PARP-1 | Cell signaling | 9542 | Rabbit |
| Bax | Cell signaling | 2772 | Rabbit |
| Bak | Calbiochem | AM04 | Mouse |
| Puma | Cell signaling | 4976 | Rabbit |
| survivin | Santa cruz | 17779 | Mouse |
| Mcl-1 | Santa cruz | 12756 | Mouse |

| Bcl-2 | Calbiochem | OP60 | Mouse |
| --- | --- | --- | --- |
| AML1 | Cell signaling | 4334 | Rabbit |
| Phospho-p70 S6 Kinase (Thr389) | Cell signaling | 9205 | Rabbit |
| p70 S6 Kinase | Cell signaling | 2708 | Rabbit |
| Phospho-4E-BP1 (Thr37/46) | Cell signaling | 2855 | Rabbit |
| 4E-BP1 | Cell signaling | 9644 | Rabbit |
| c-MYC | Genetex | 109636 | Rabbit |
| LDHA | Genetex | 101416 | Rabbit |
| Phospho-PKM2 (Tyr105) | Cell signaling | 3827 | Rabbit |
| PKM2  HSP90 | Cell signaling  Genetex | 3198  101448 | Rabbit  Rabbit |
| Anti-puromycin | Merck | MABE343 | Mouse |

**Supplementary table 2.**

Primer sequence of RT-qPCR

| gene | Primer sequence (5’-3’) |
| --- | --- |
| Apoptosis-related genes | |
| *Survivin* | F - CATCTCTACATTCAAGAACTGG  R - GGTTAATTCTTCAAACTGCTTC |
| *BBC3* | F - CCTGGAGGGTCCTGTACAATCT  R - GCACCTAATTGGGCTCCATCT |
| Glycolysis-related genes | |
| *GLUT1* | F - AGGTGATCGAGGAGTTCTAC  R - TCAAAGGACTTGCCCAGTTT |
| *LDHA* | F - AGCCCGATTCCGTTACCT  R - CACCAGCAACATTCATTCCA |
| *PKM2* | F - CCACTTGCAATTATTTGAGGAA  R - GTGAGCAGACCTGCCAGACT |
| AML1-ETO | |
| *AML1-ETO* | F - ATGACCTCAGGTTTGTCGGTCG  R - TGAACTGGTTCTTGGAGCCTCCT |
| AML1-ETO target genes | |
| *NFE2* | F - CCAAGGTGTGTTCAAAGAGGC  R - GGAGCCGAGTCAGGGAAGAC |
| *S100A8* | F - CGAGCTGGAGAAAGCCTTGA  R - GACGTCTGCACCCTTTTTCC |
| *LYZ* | F - TGCTGCAAGATAACATCGCTG  R - CCATGCTCTAATGCCTTGTGG |
| *RNASE2* | F - CCTCACAACTCCAAGTCCACAG  R - TACTGTGGAGGGTCTCGTCGTT |
| *CD24* | F - CTCCTACCCACGCAGATTTATTC  R - AGAGTGAGACCACGAAGAGAC |
| *CTSG* | F - TCCTGGTGCGAGAAGACTTTG  R - GGTGTTTTCCCGTCTCTGGA |
| *IGFBP7* | F - GAAGTAACTGGCTGGGTGCTG  R - GCTGATGCTGAAGCCTGTCC |
| *CXCR4* | F - ACTACACCGAGGAAATGGGCT  R - CCCACAATGCCAGTTAAGAAGA |
| *SRGN* | F - AGGTTATCCTACGCGGAGAG  R - GTCTTTGGAAAAAGGTCAGTCCT |
| Internal control | |
| *Actin* | F - CACCATTGGCAATGAGCGGTTC  R - AGGTCTTTGCGGATGTCCACGT |
