# Supplementary material for: Repurposing cabozantinib with therapeutic potential in KIT-driven t(8;21) acute myeloid leukaemias
Source: Cancer Gene Ther. 2021 Apr 8;29(5):519–32. doi: 10.1038/s41417-021-00329-1 (PMC9113930; doi:10.1038/s41417-021-00329-1)
Supplement: Supplementary file 2 — Data Set 1 [file 41417_2021_329_MOESM2_ESM.docx]

**Uncropped Western blotting figures**

**Figure 1C.**


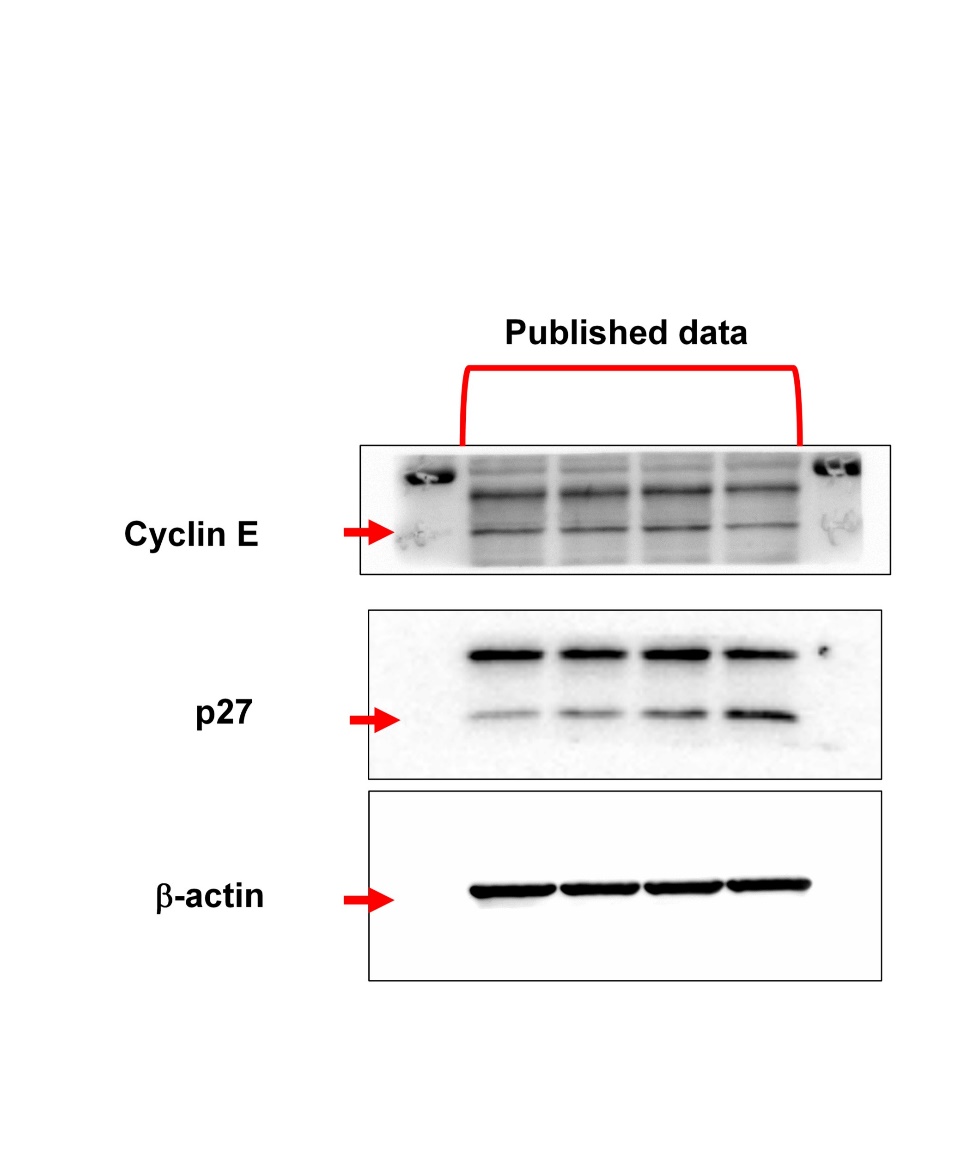


**Figure 1E.**

**
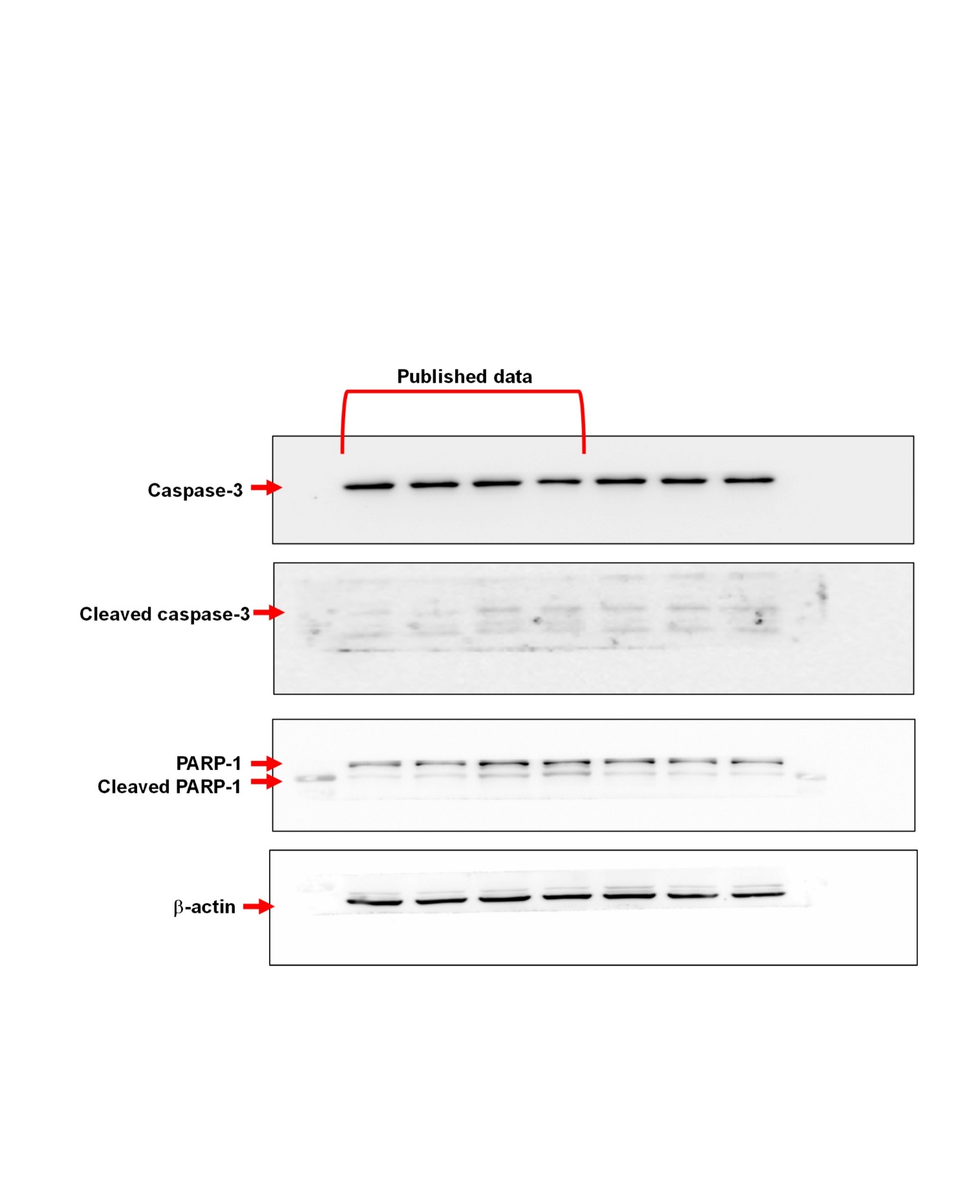
**

**Figure 1F.**

**
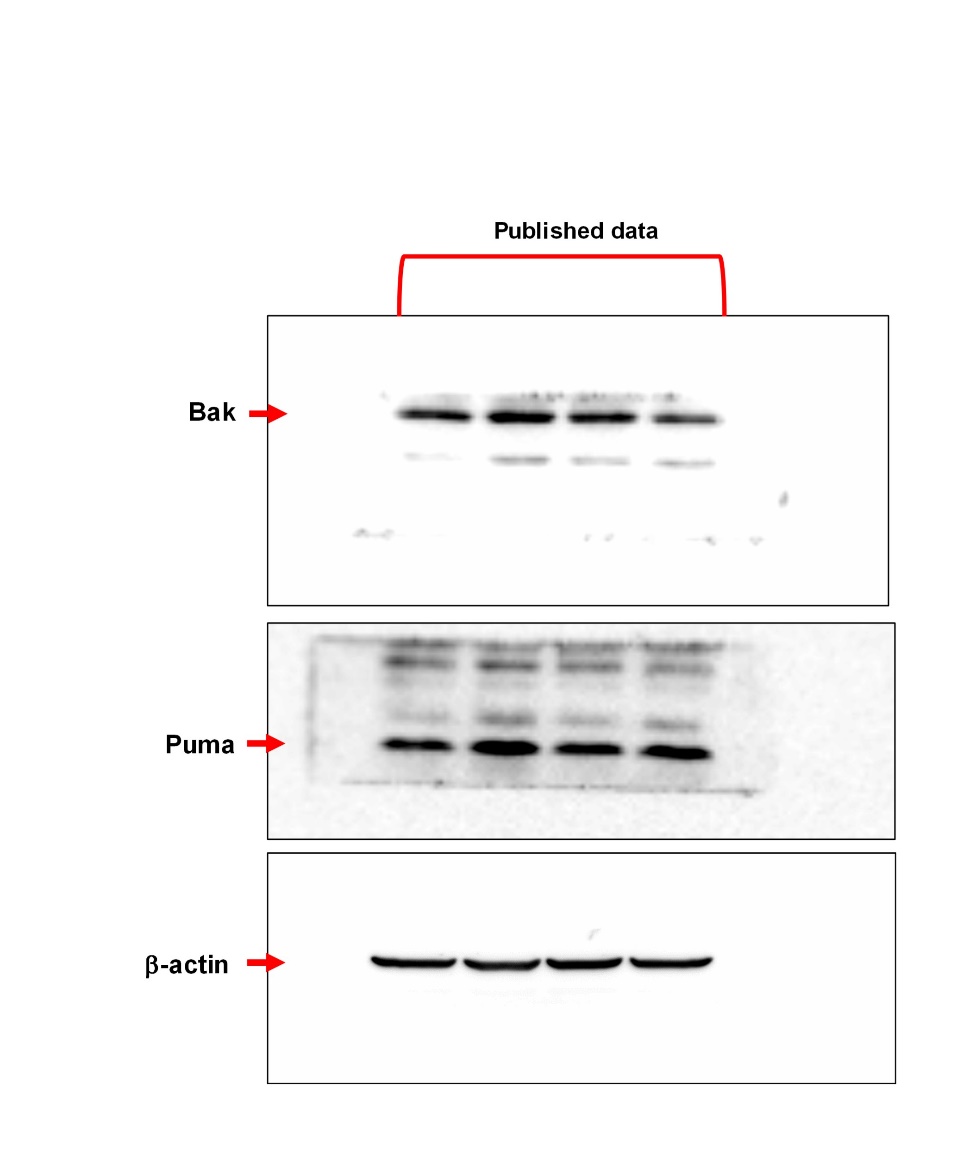
**

**
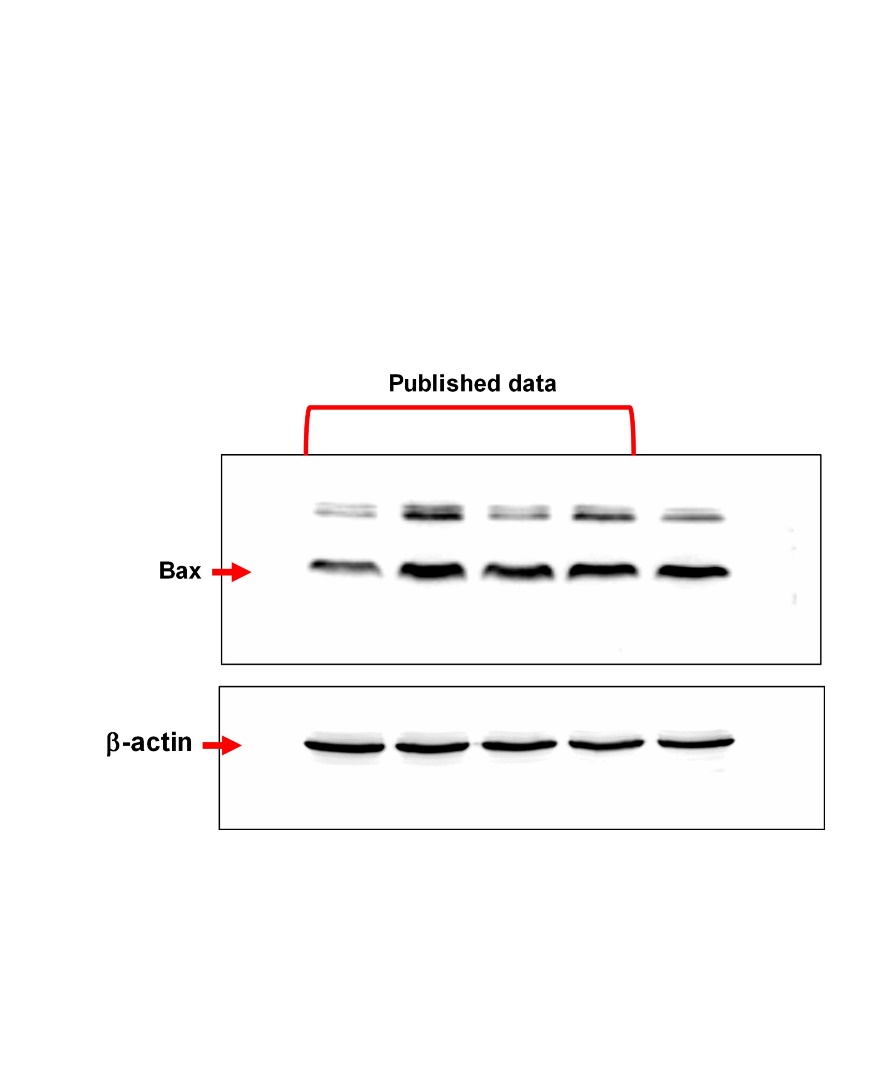
**

**
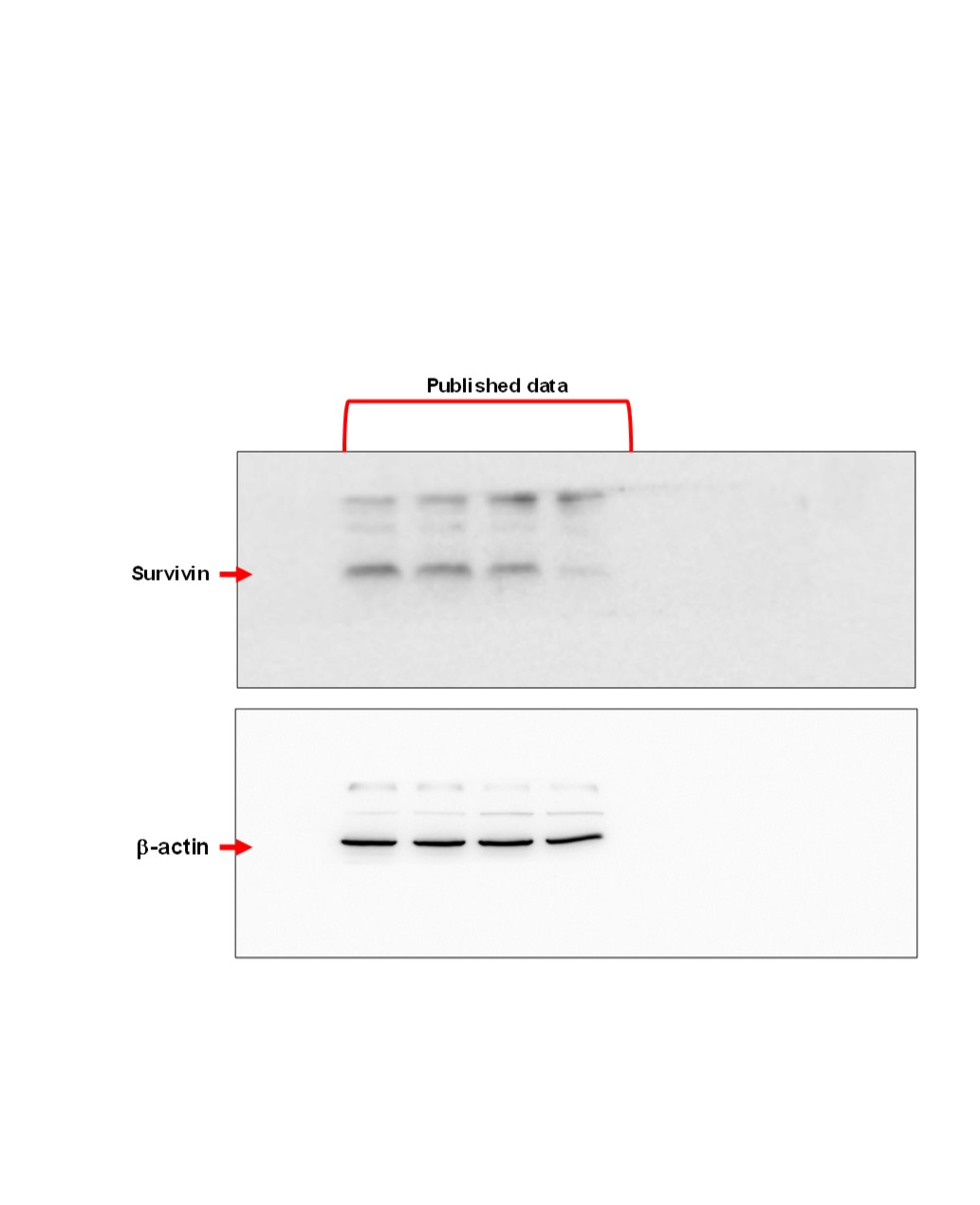
**

**
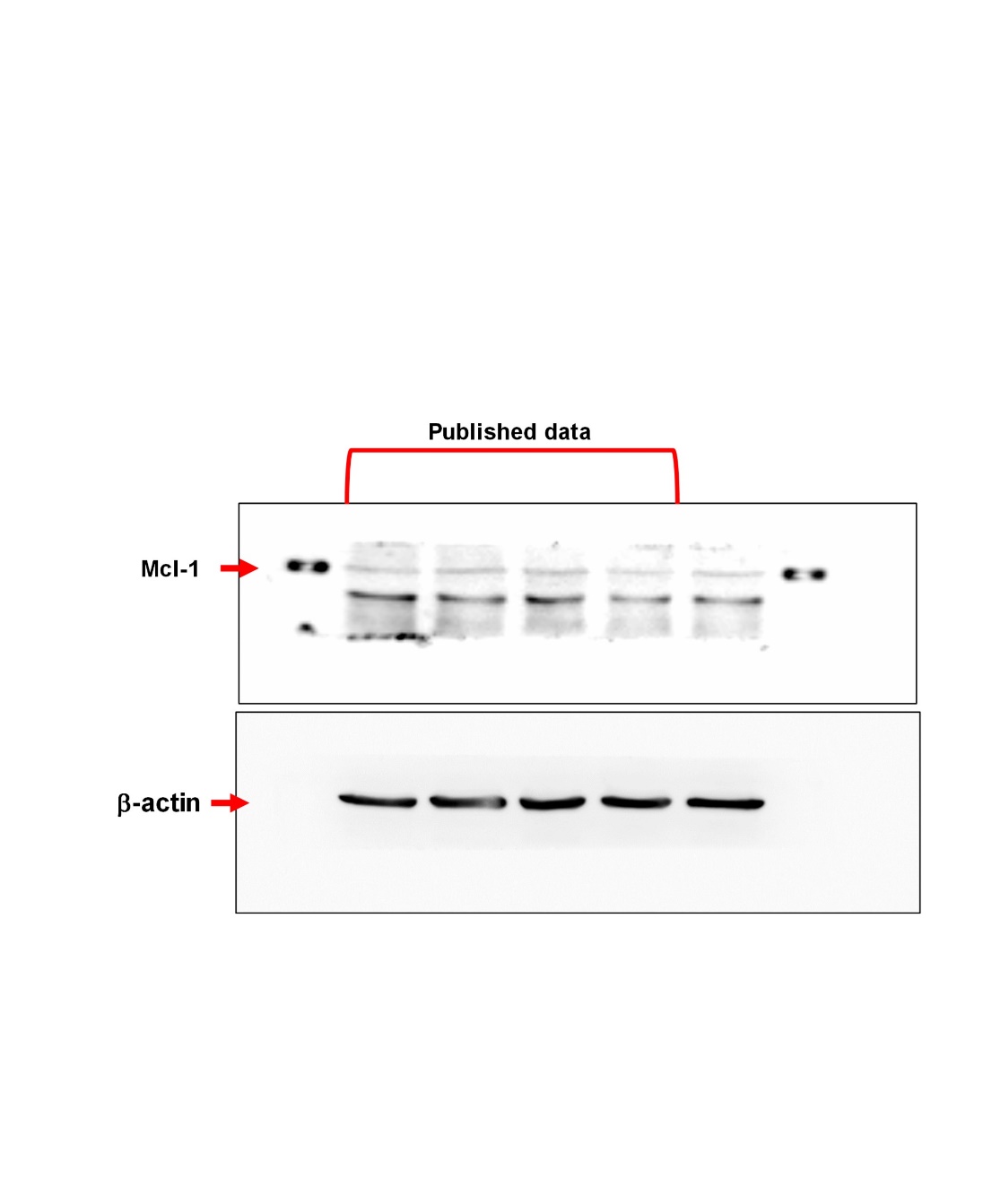

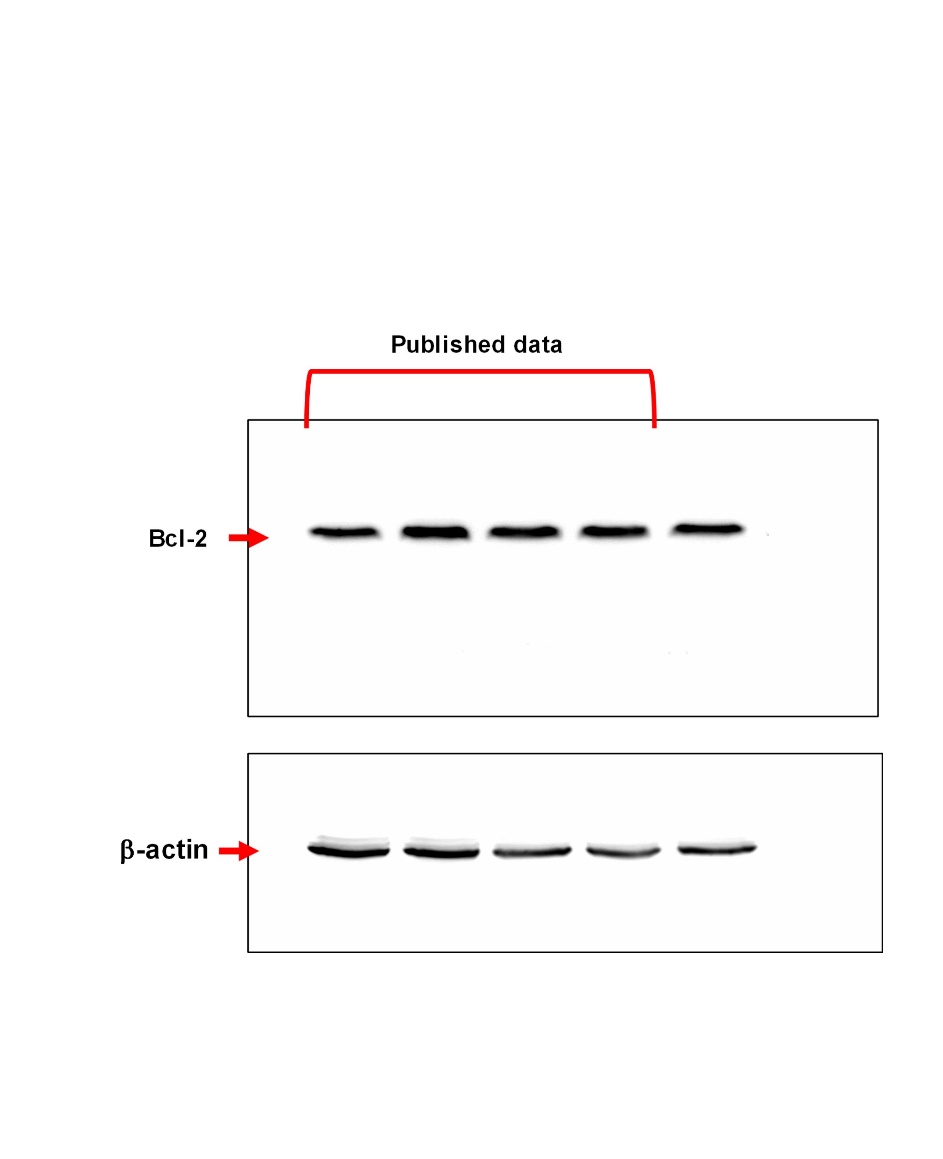
**

**Figure 2A.**

**
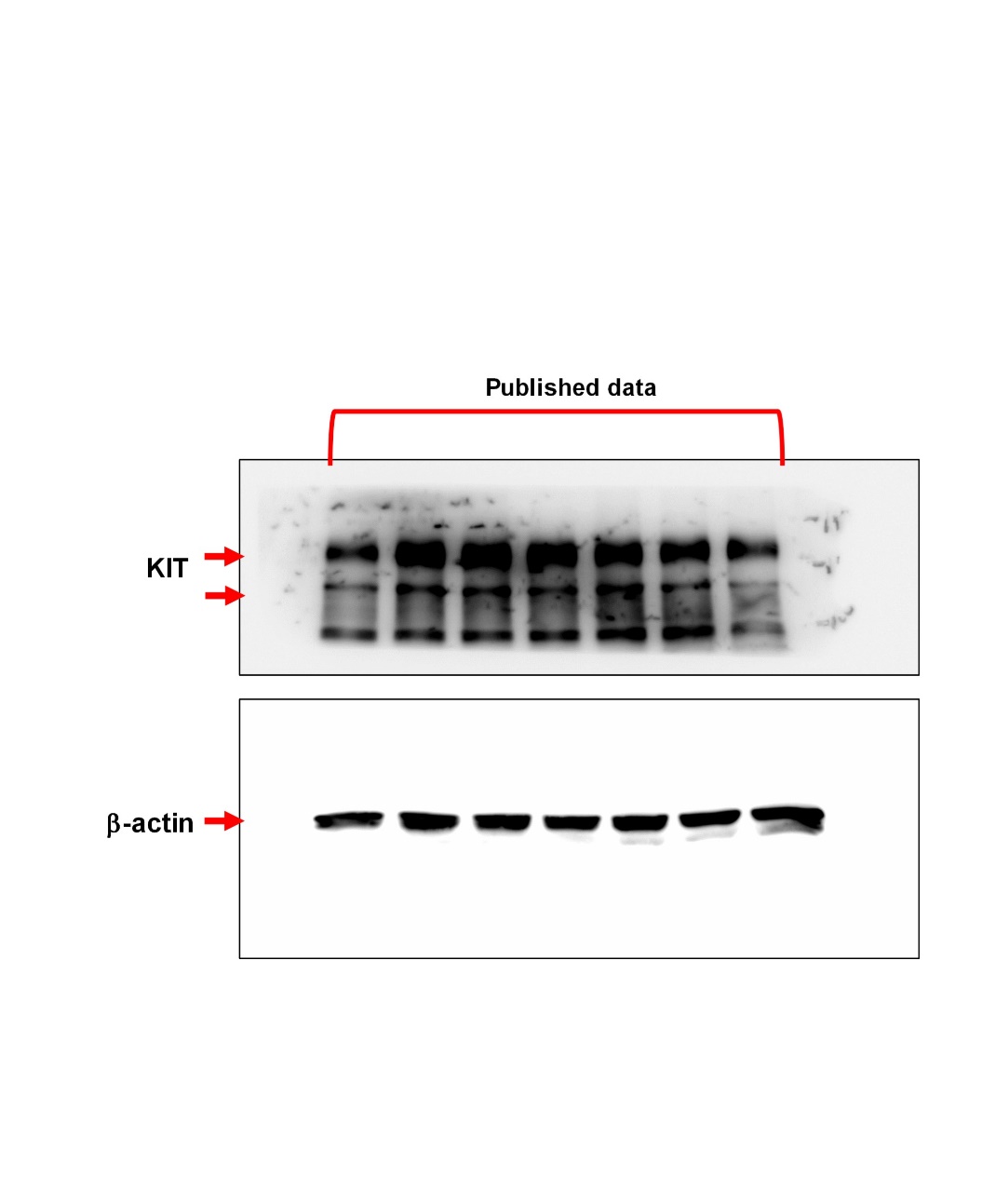
**

**
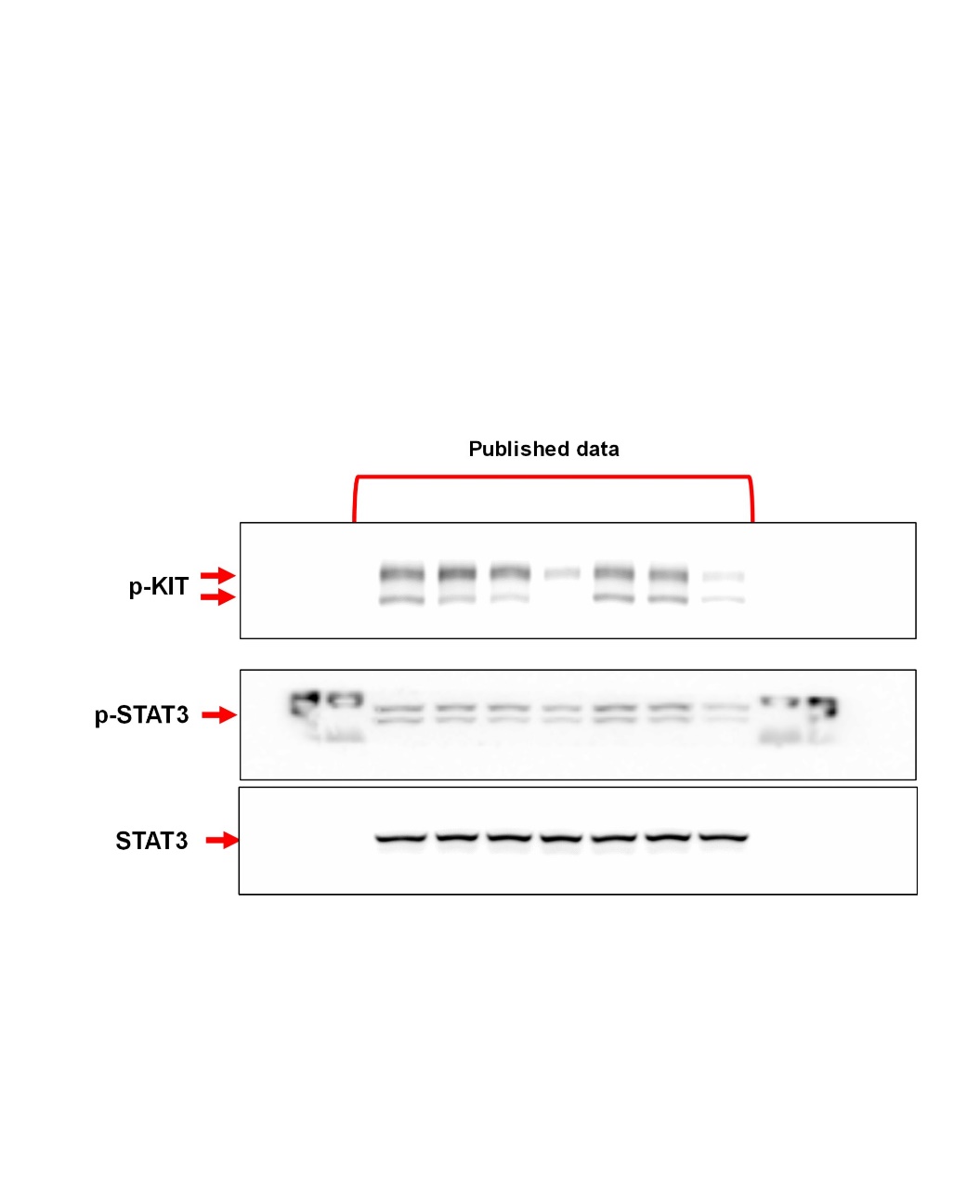
**

**
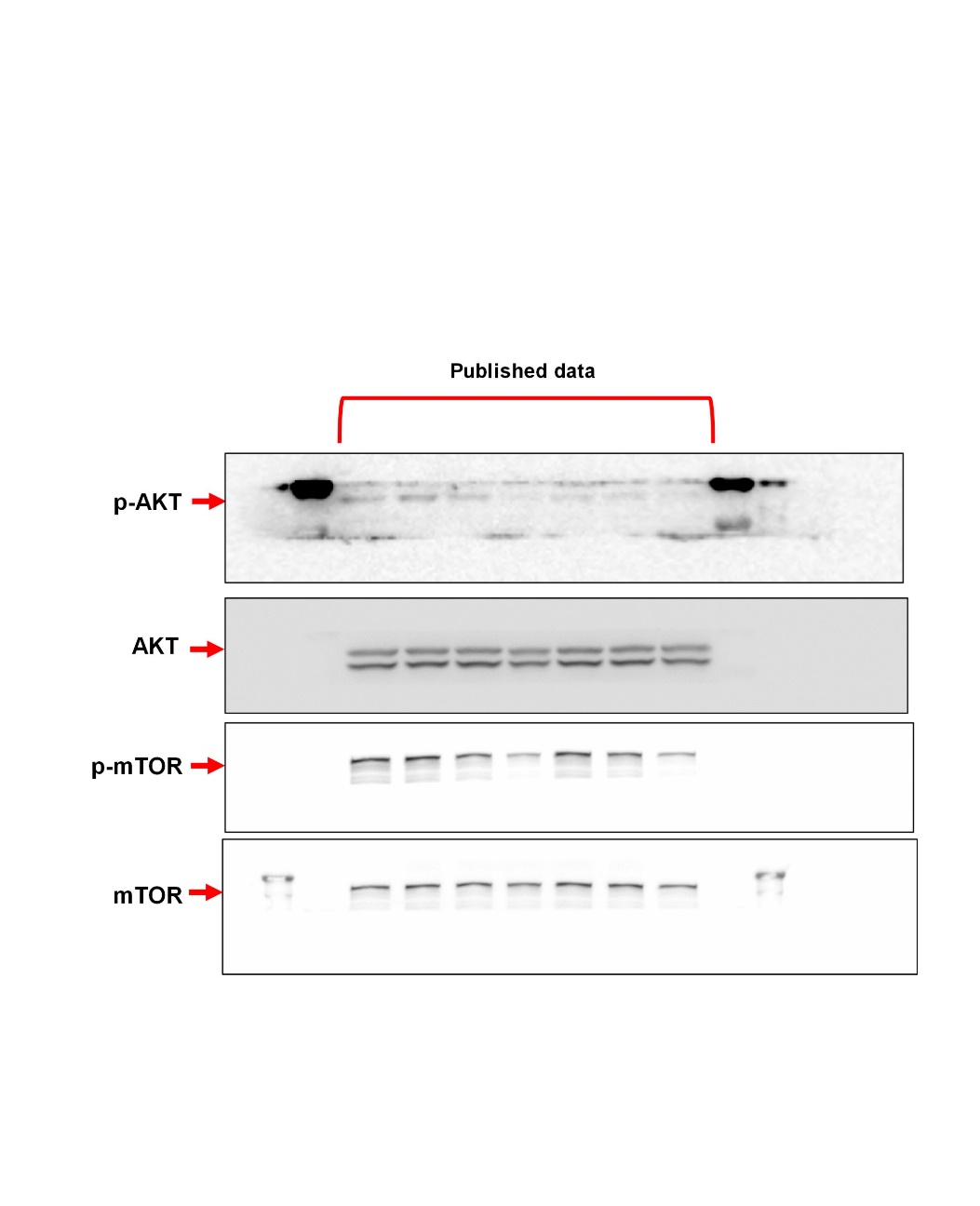
**

**
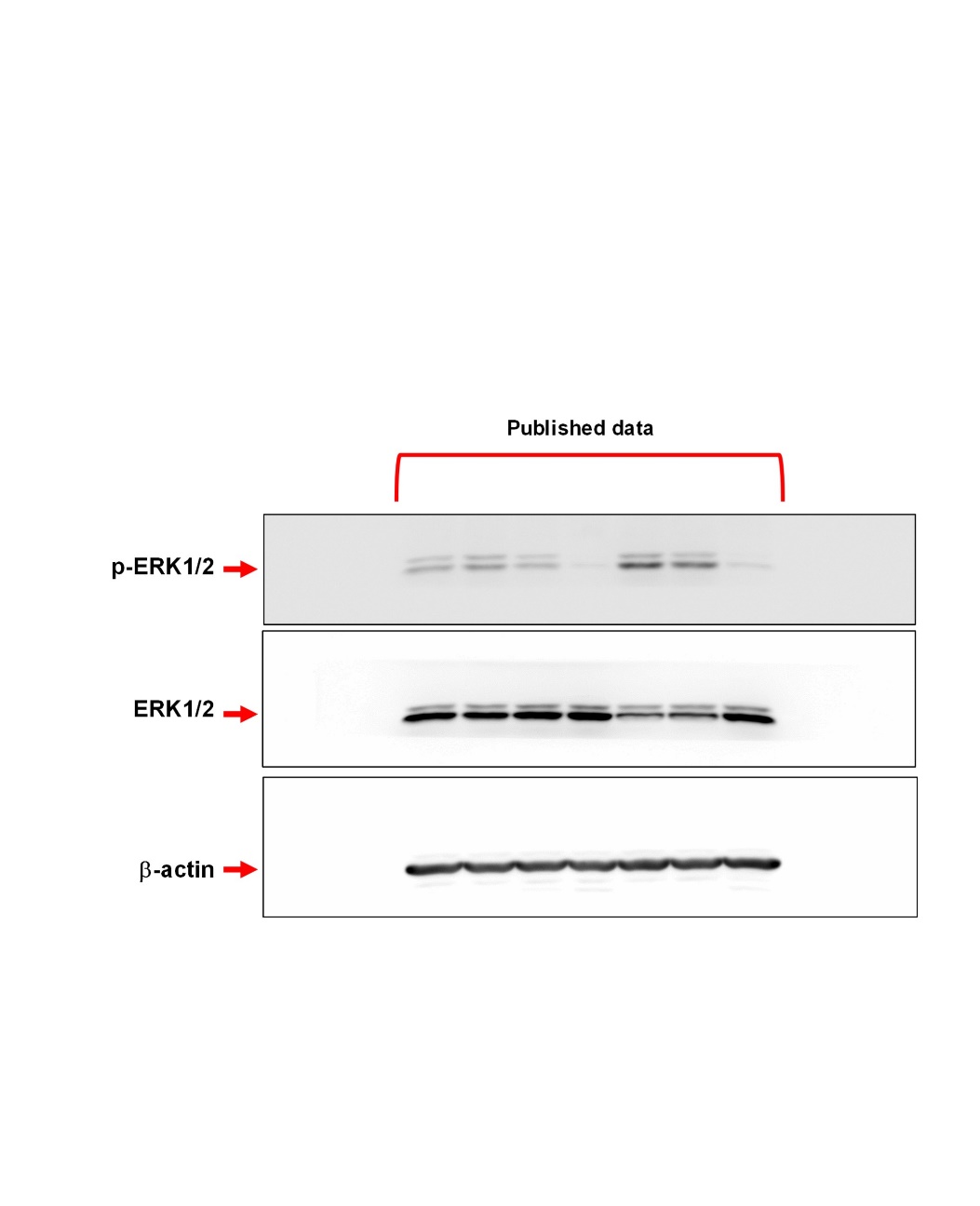
**

**Figure 2B.**

**
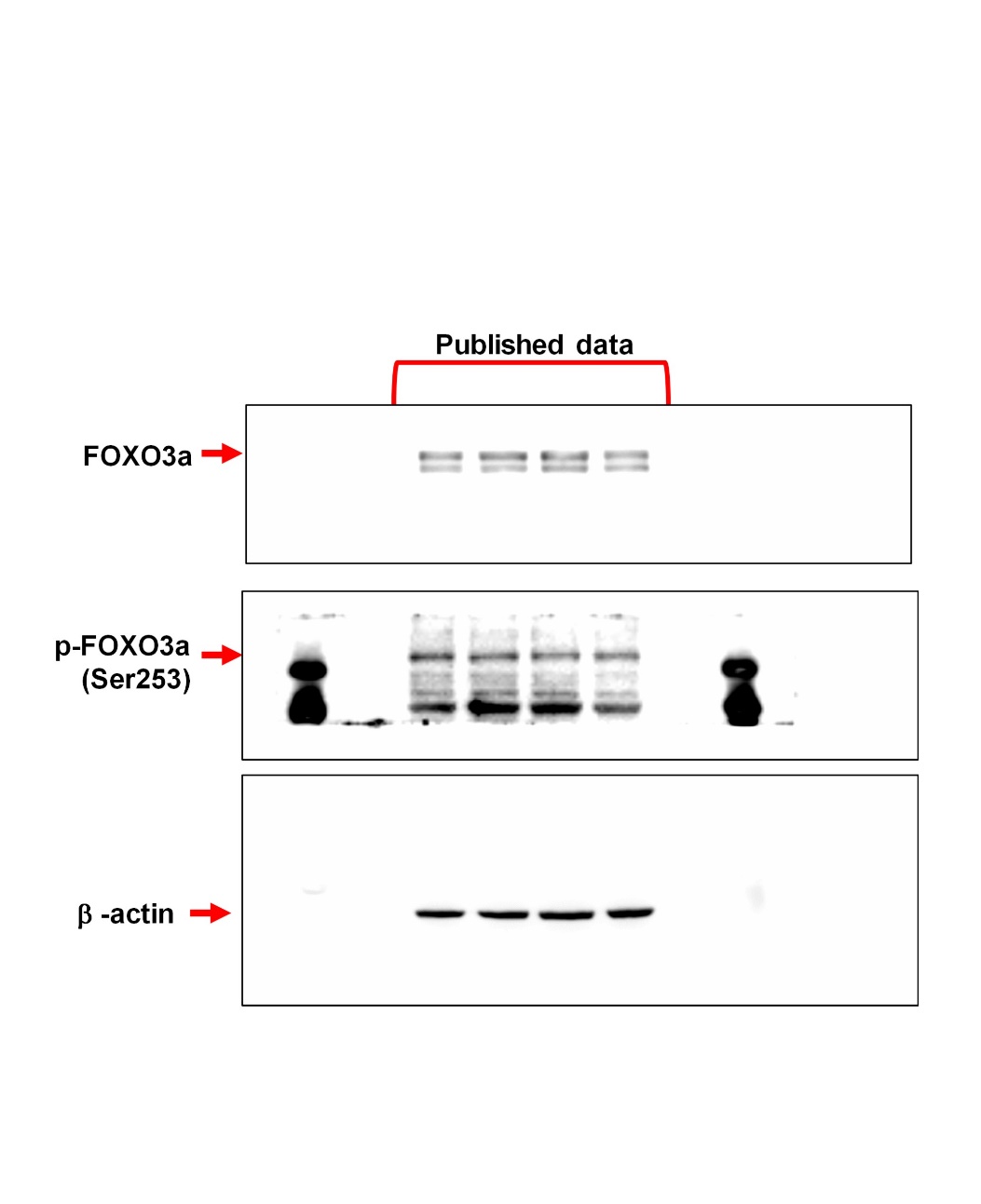
**

**Figure 2C.**

**
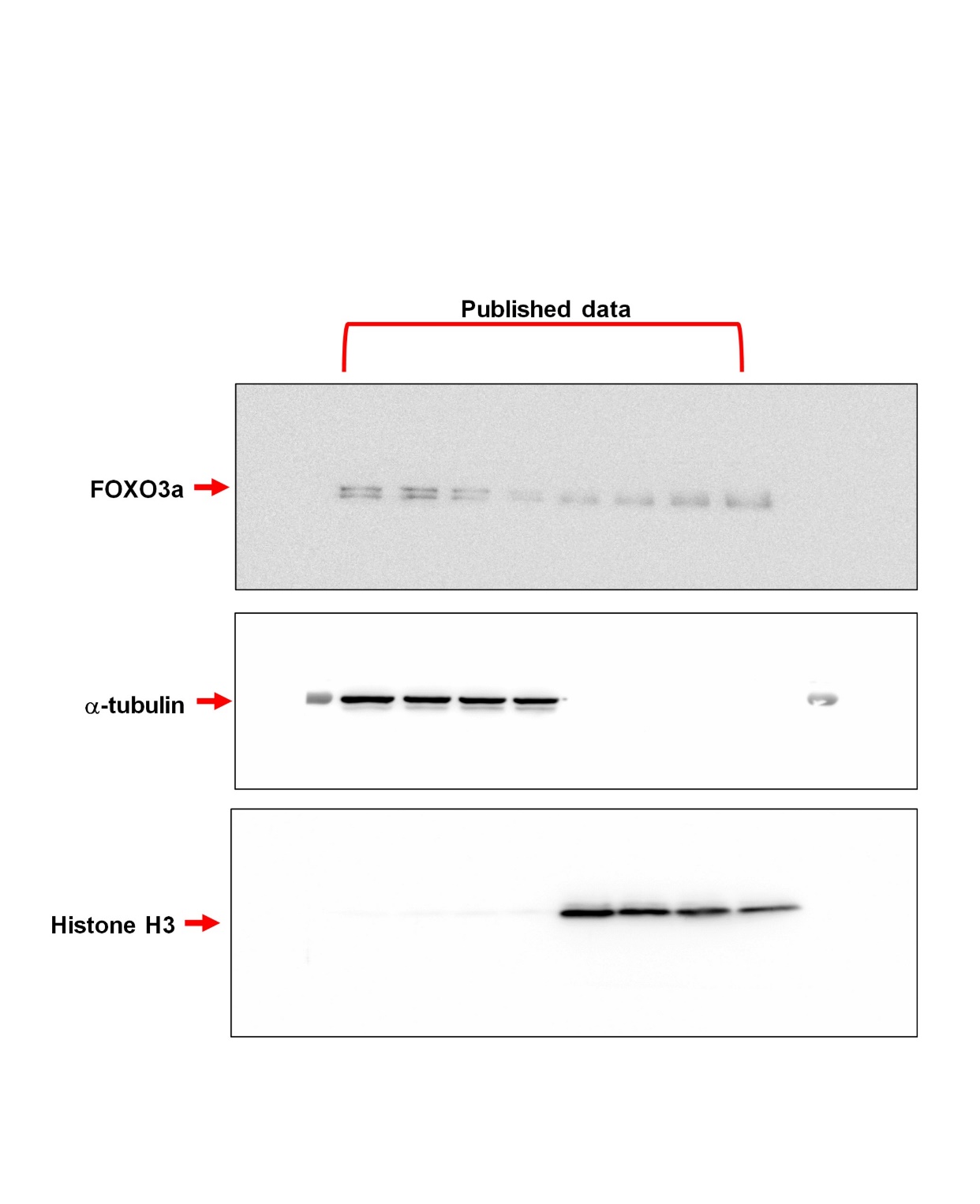
**

**Figure 3A.**

**
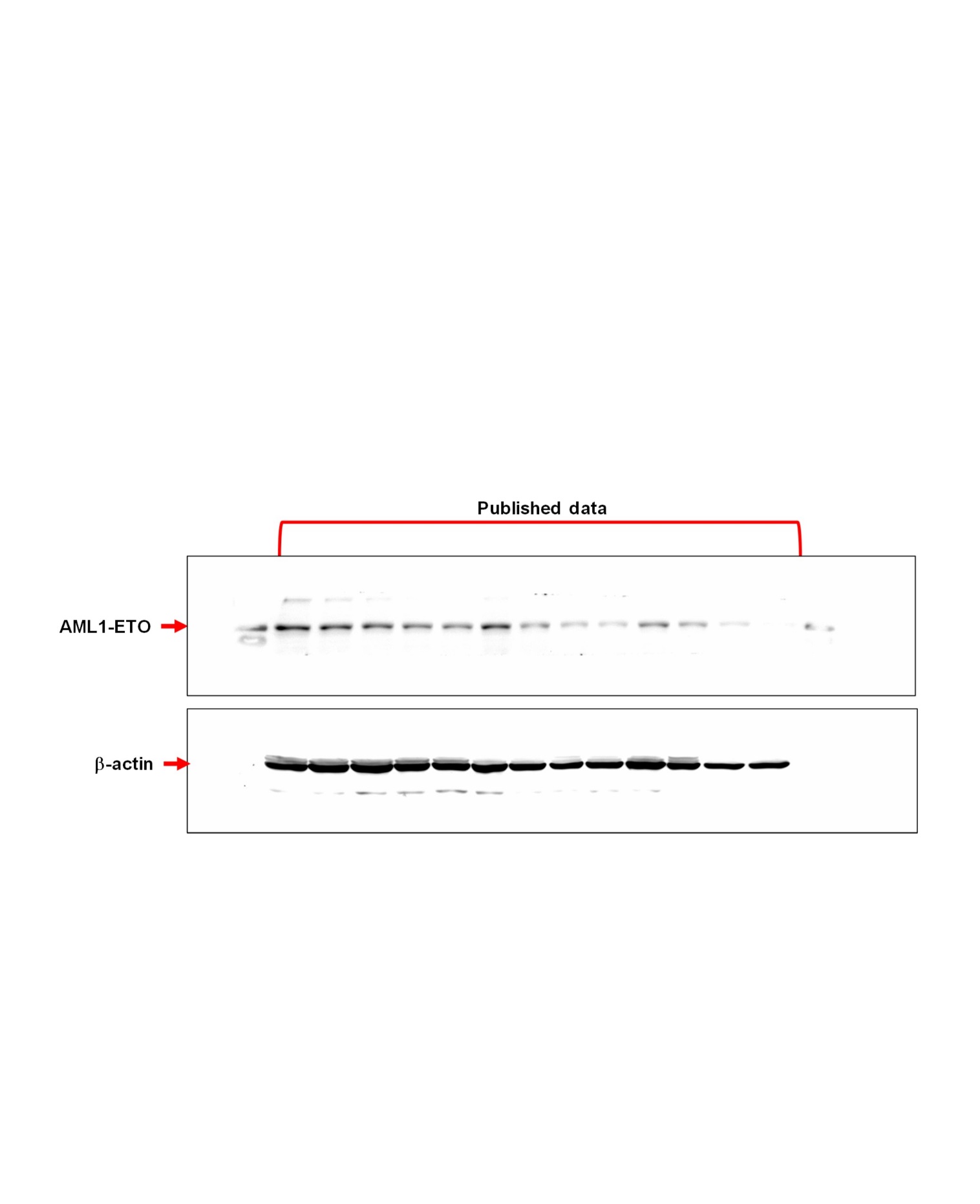
**

**Figure 3B.**

**
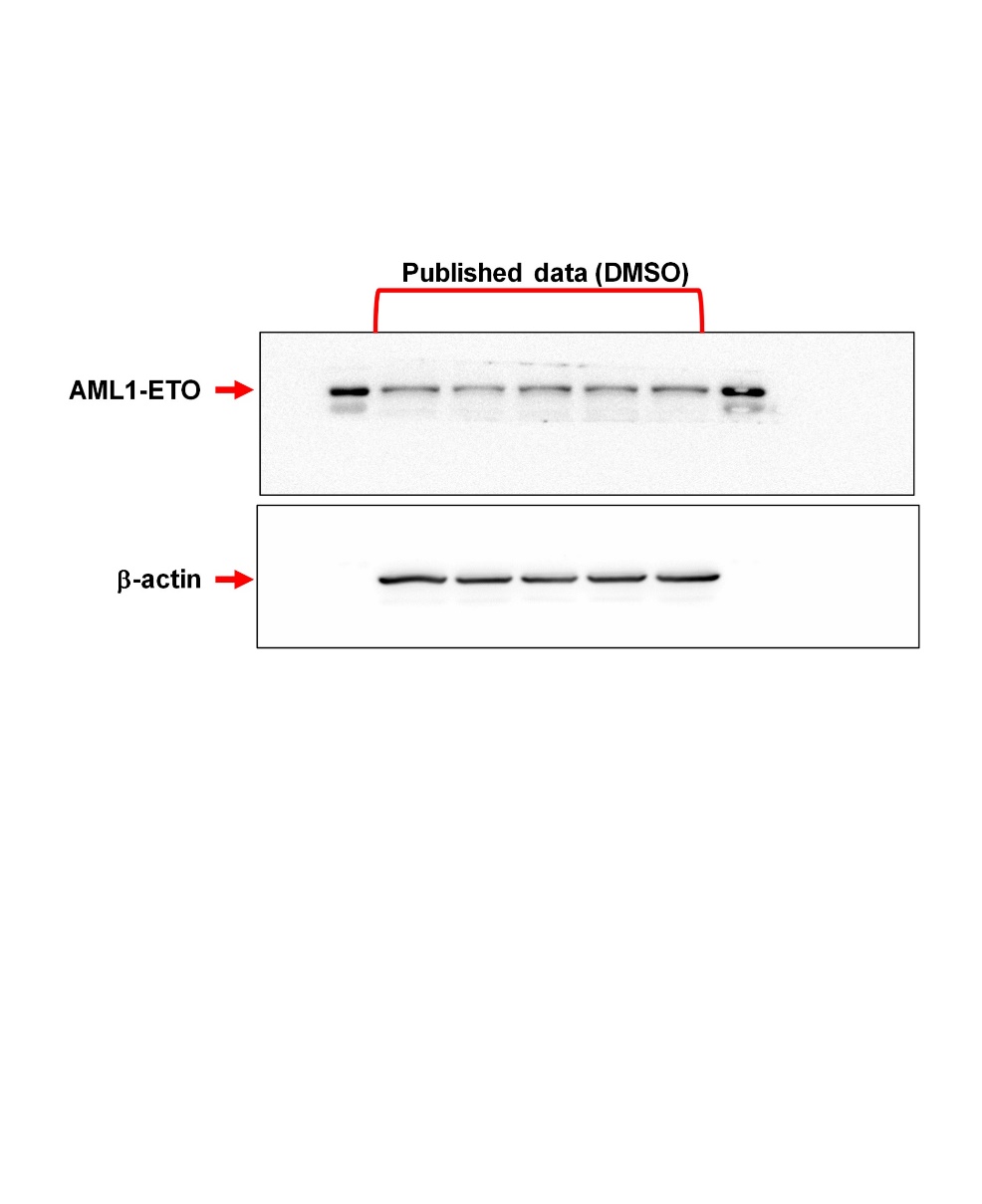
**

**
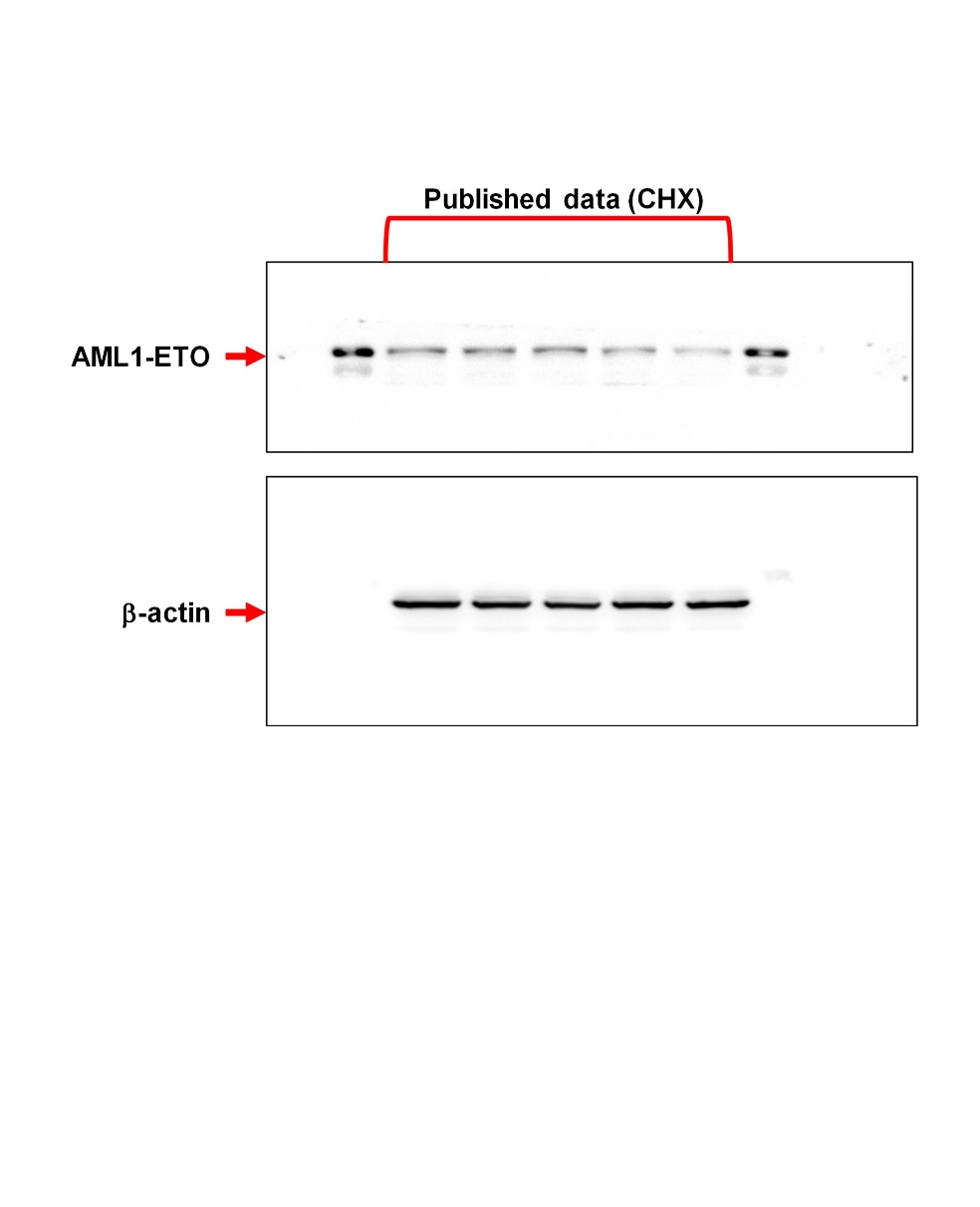
**

**
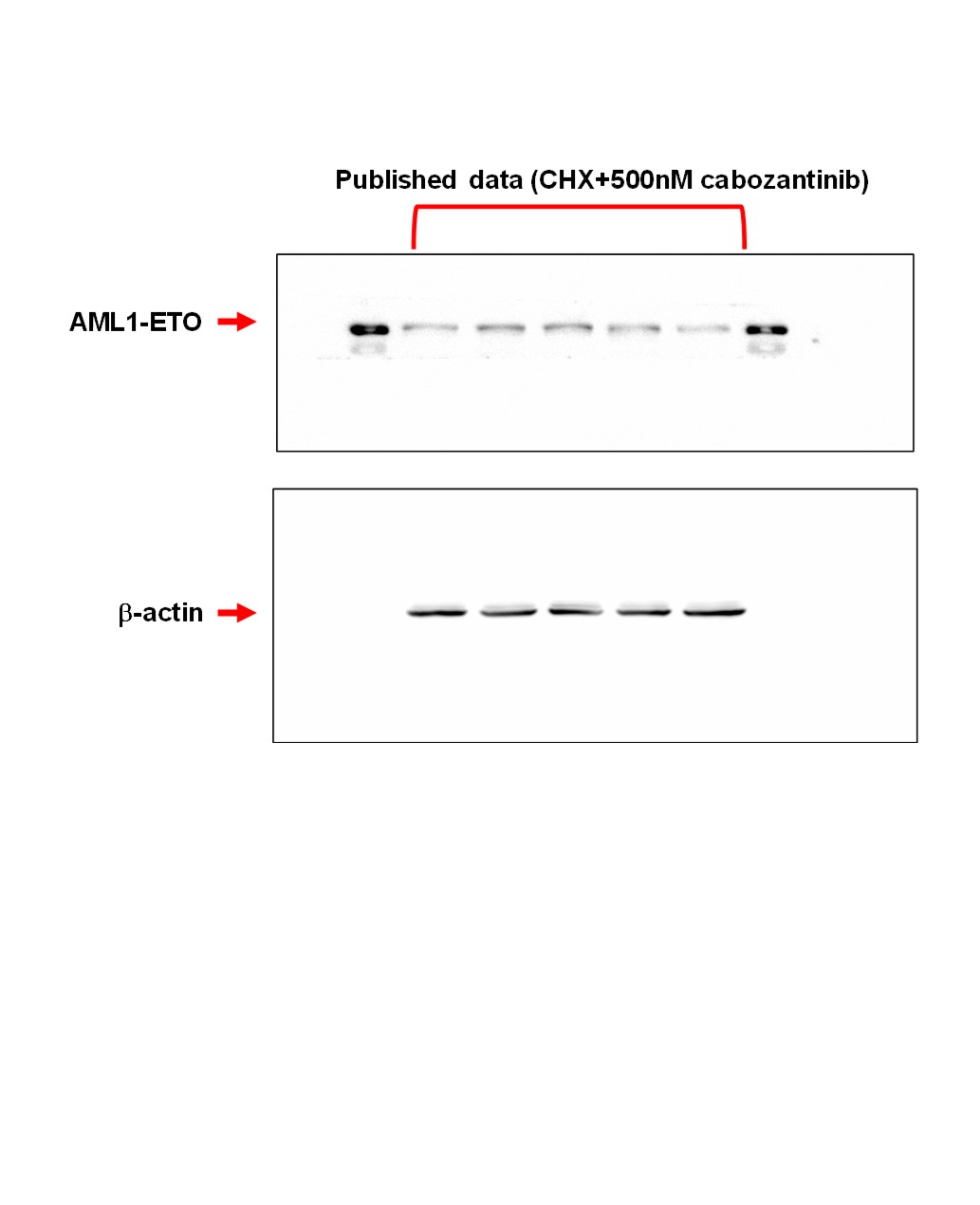
**

**
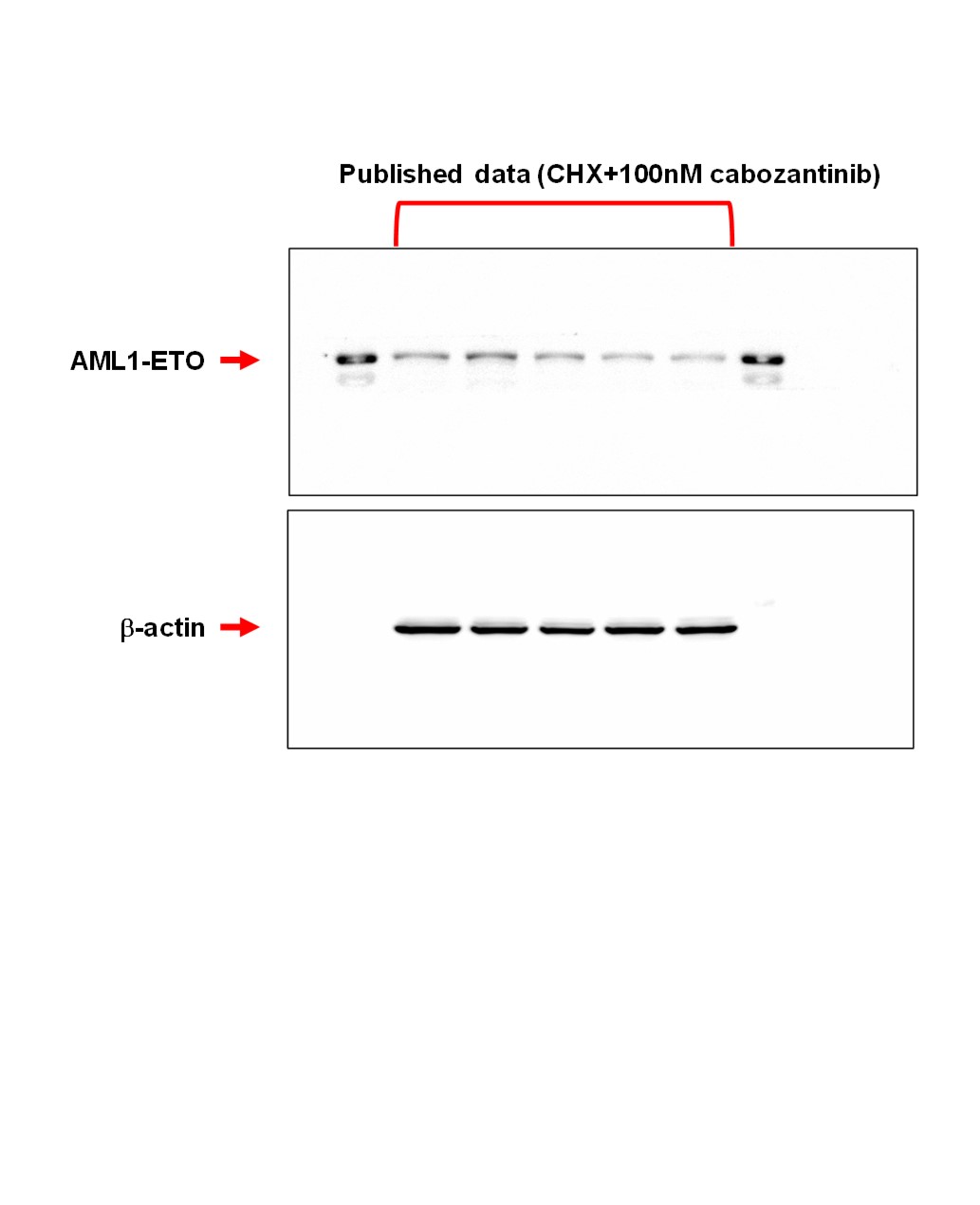
**

**Figure 3C.**

**
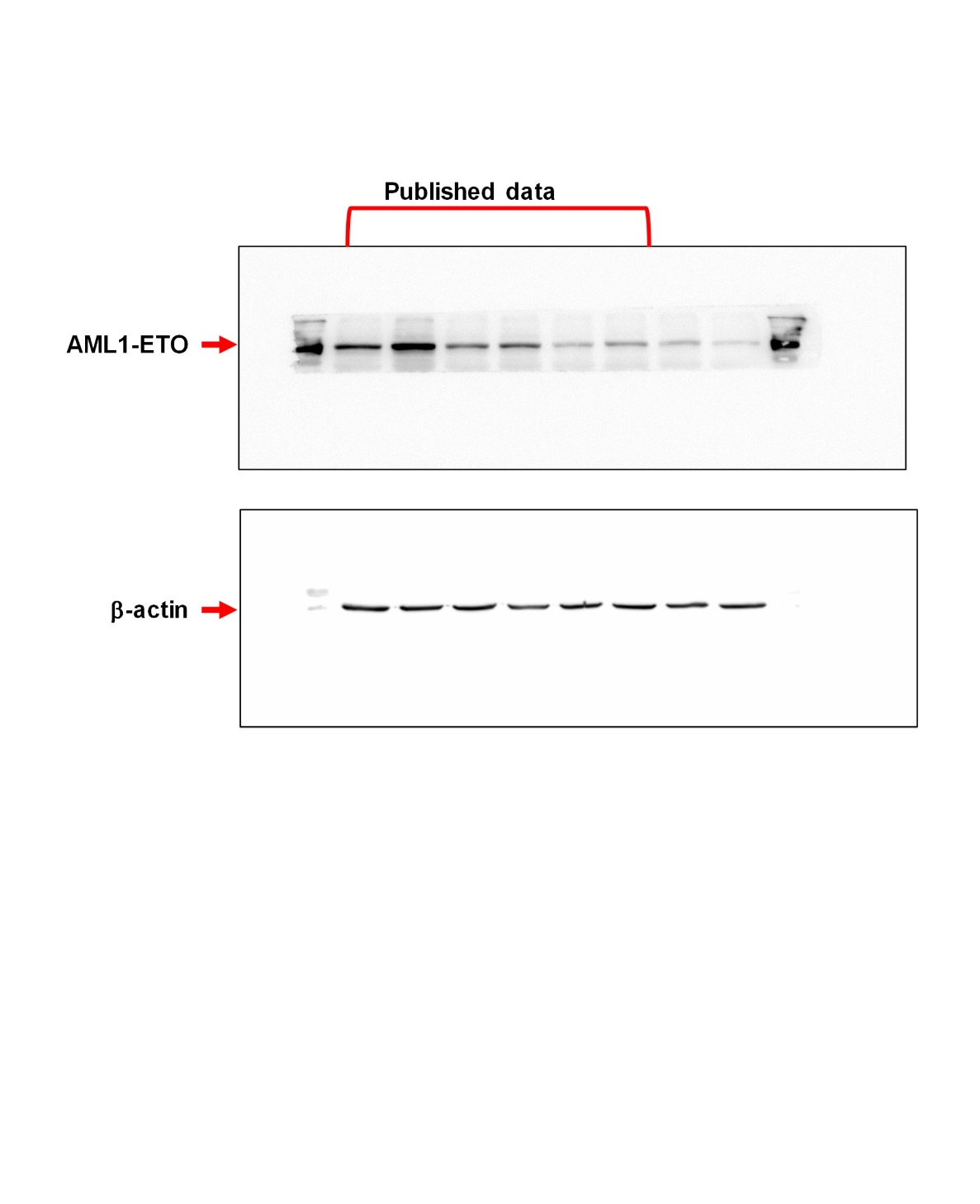
**

**Figure 3D.**

**
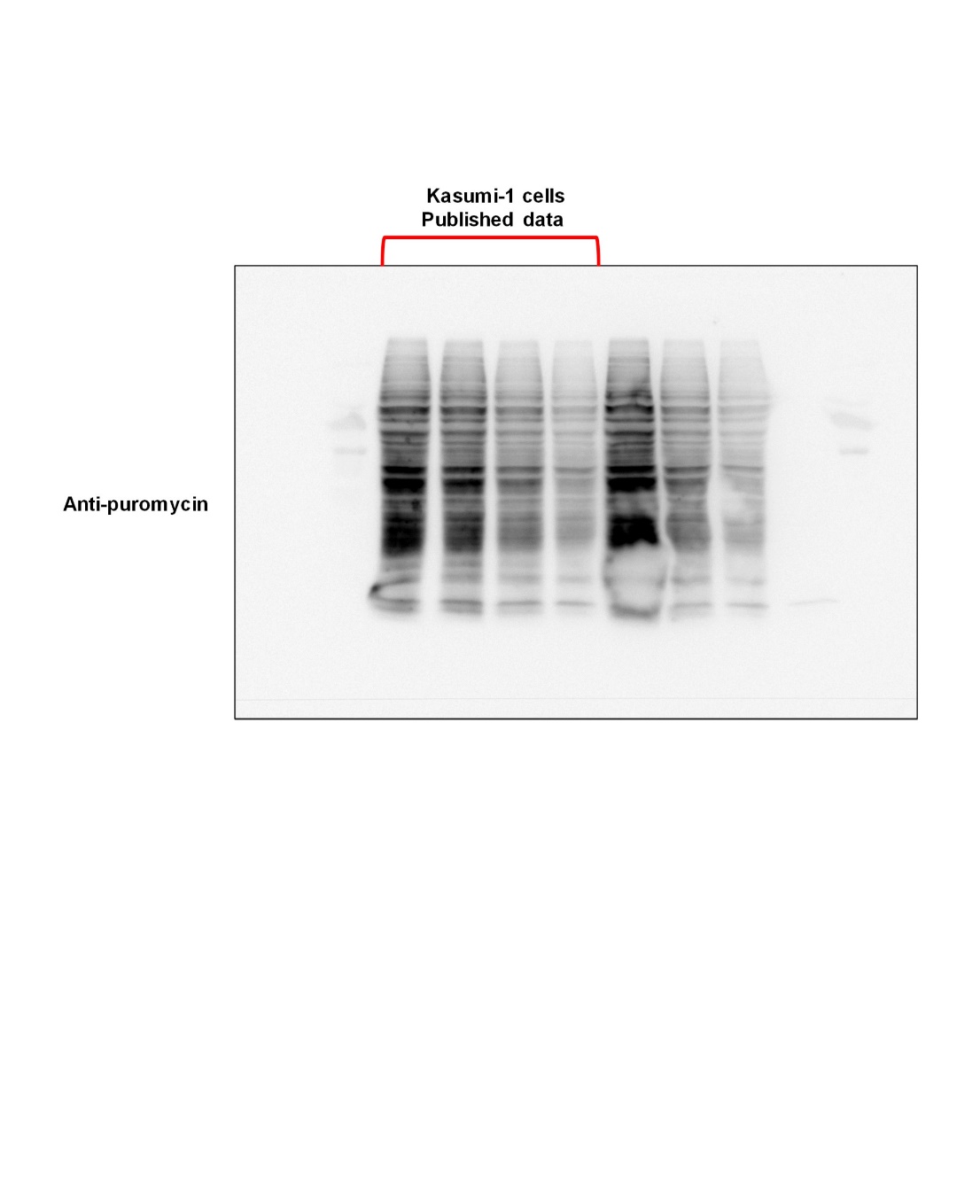

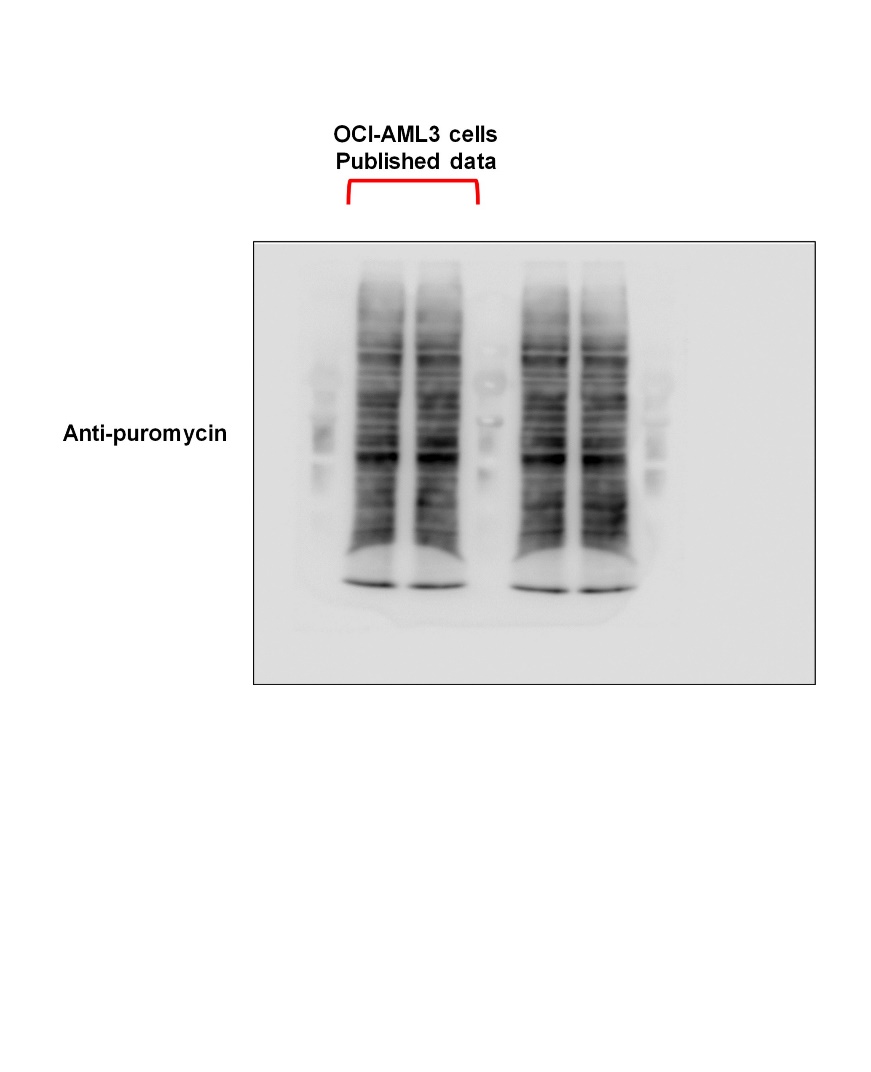
**

**
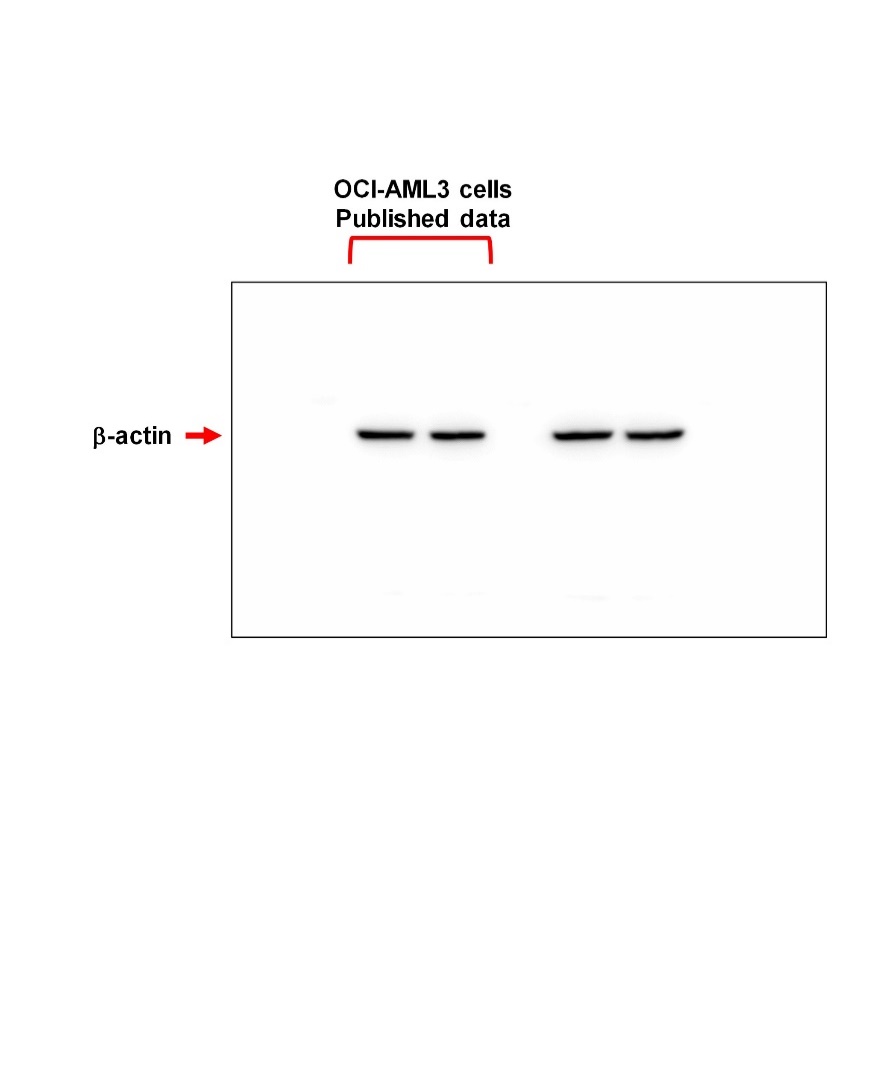

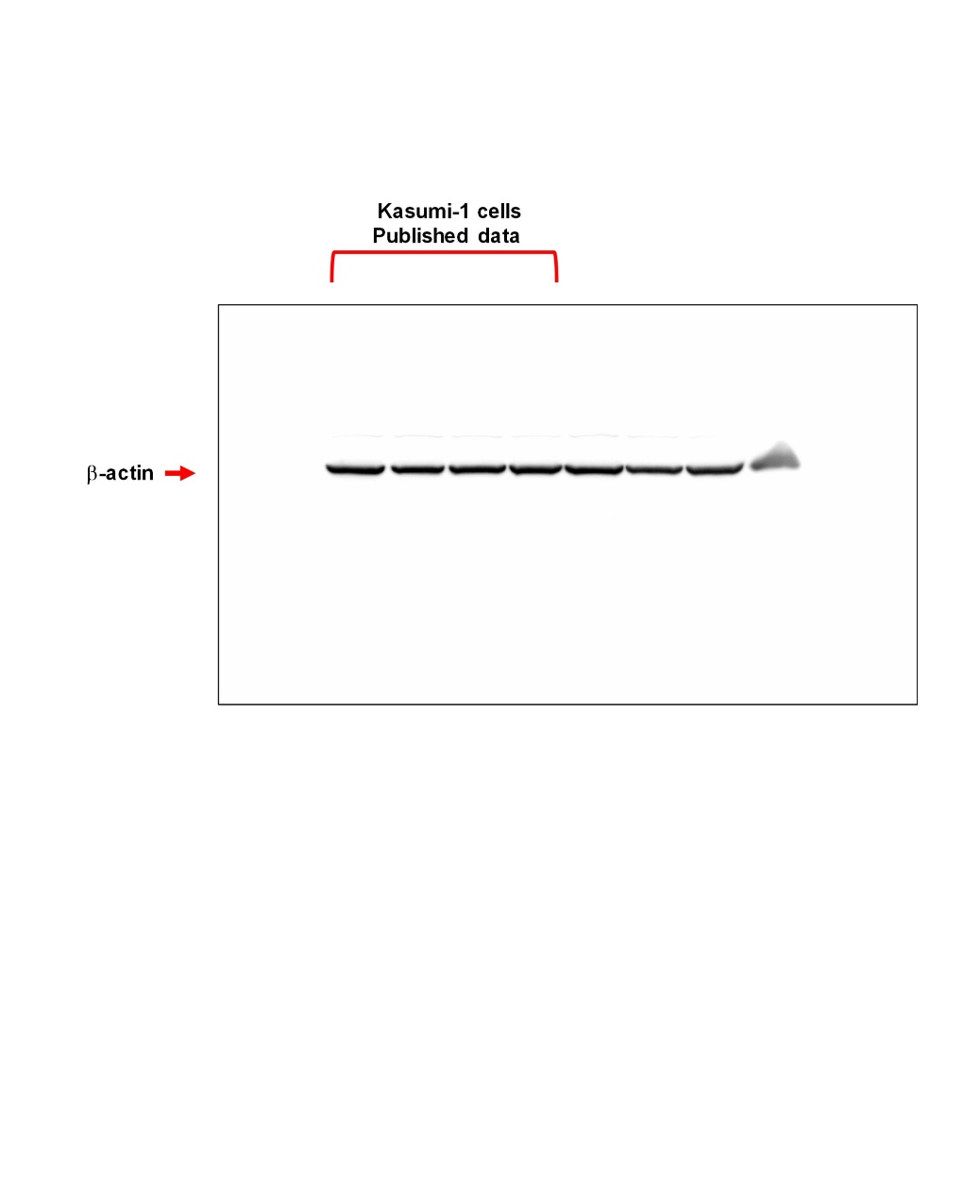
**

**
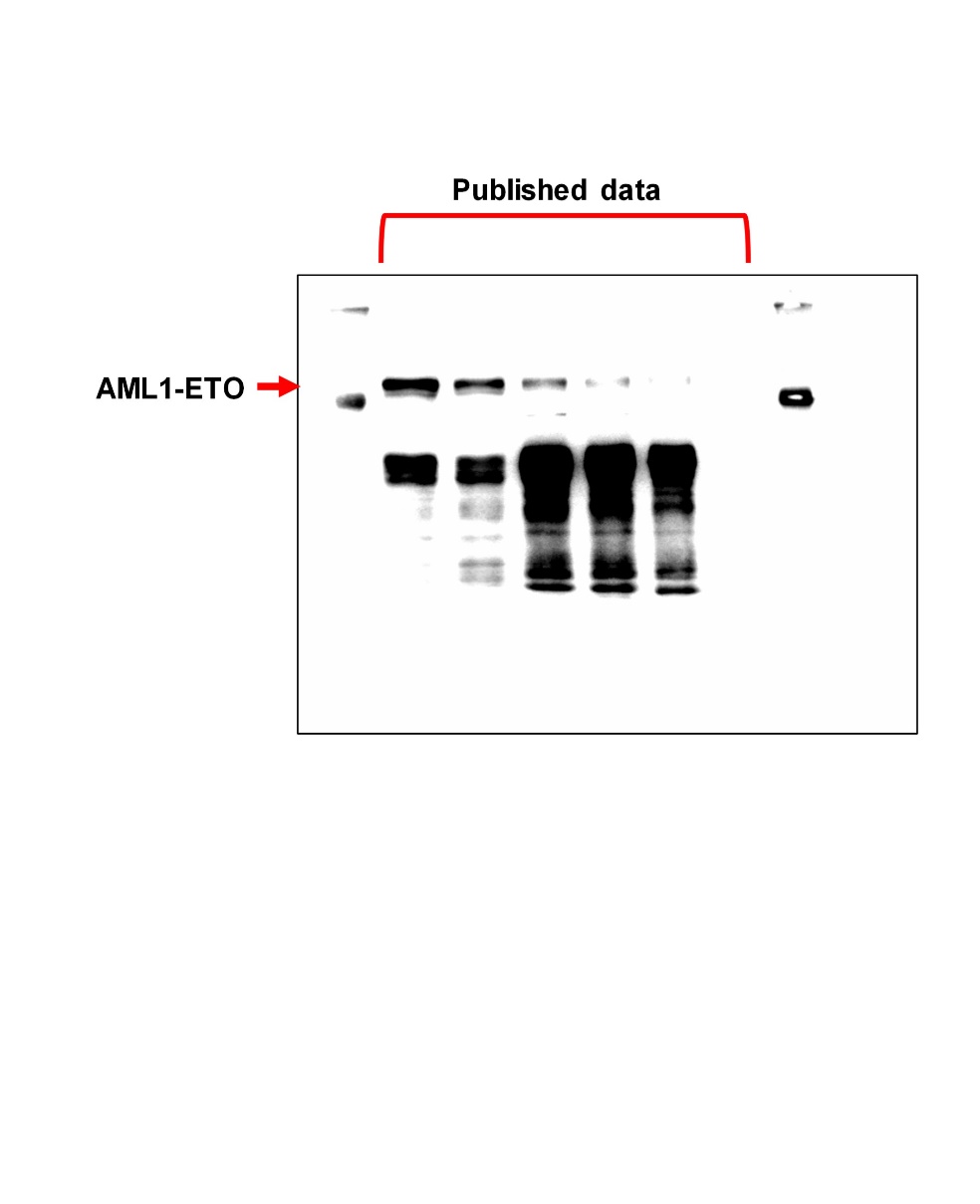
Figure 3E.**

**
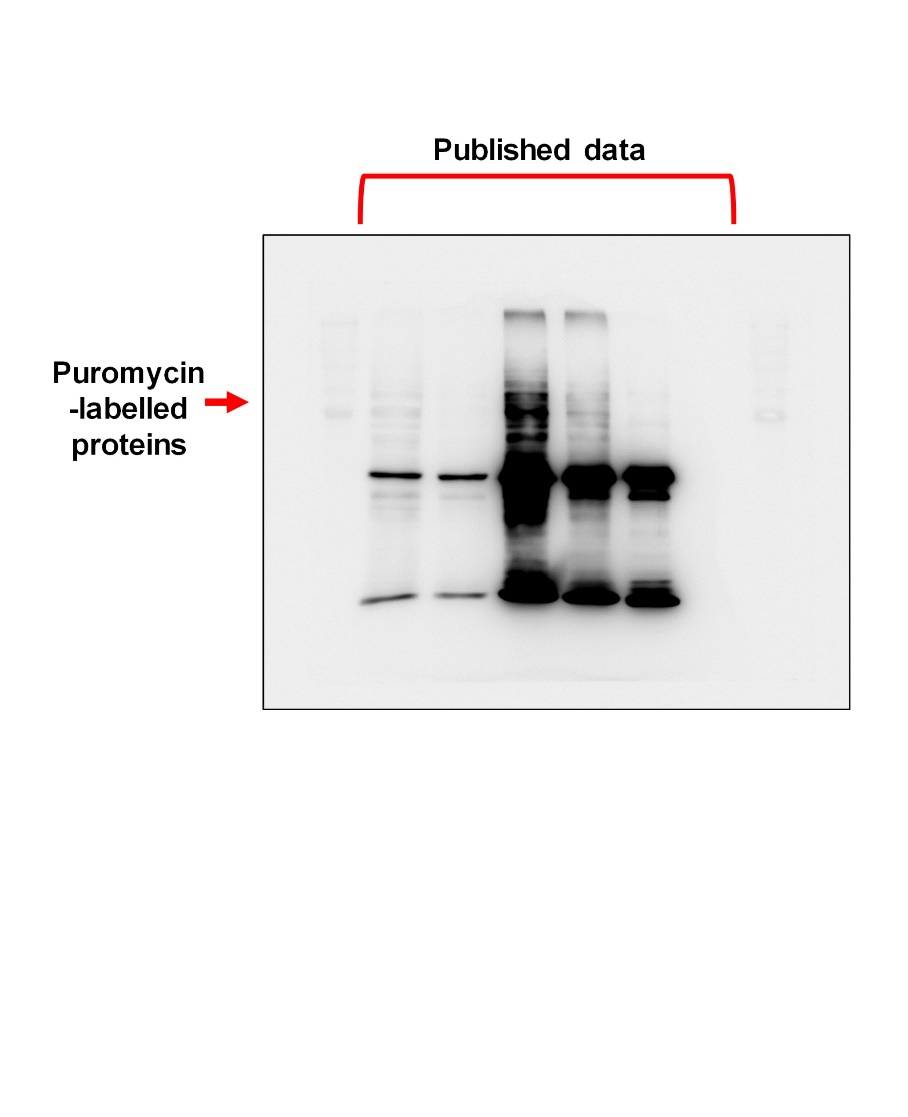
**

**Figure 4E.**

**
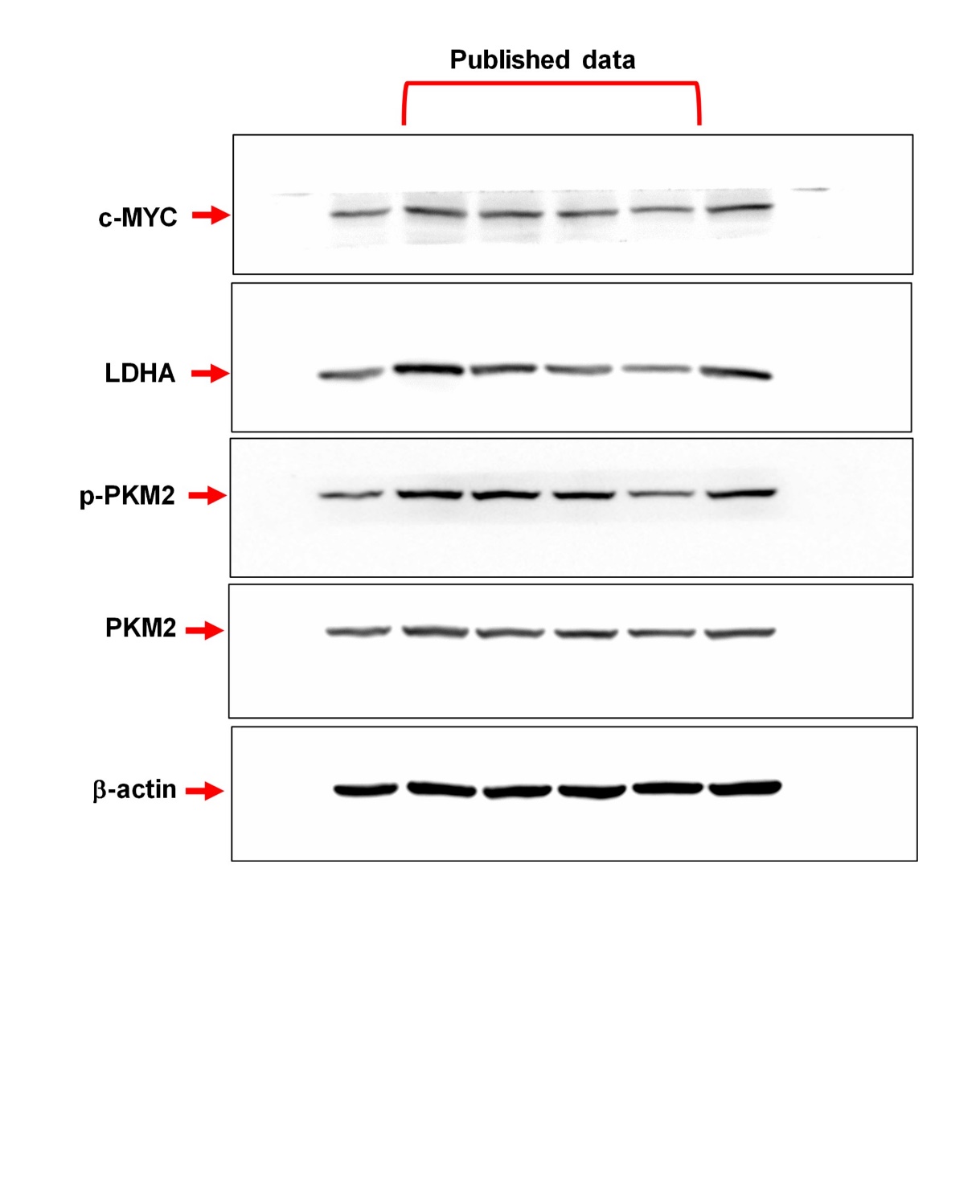
**

**Figure 5A.**

**
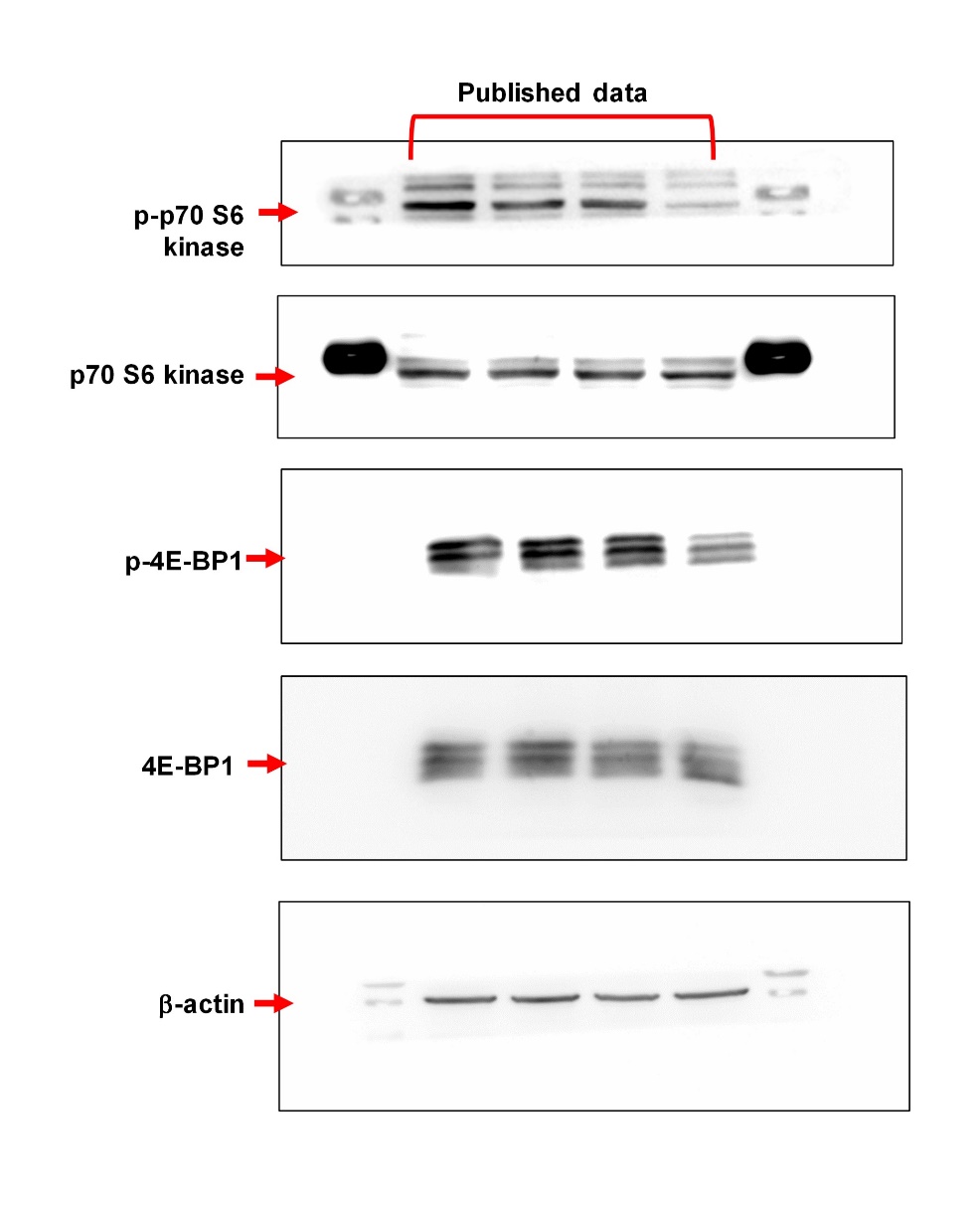
**

**Figure 6E.**

**
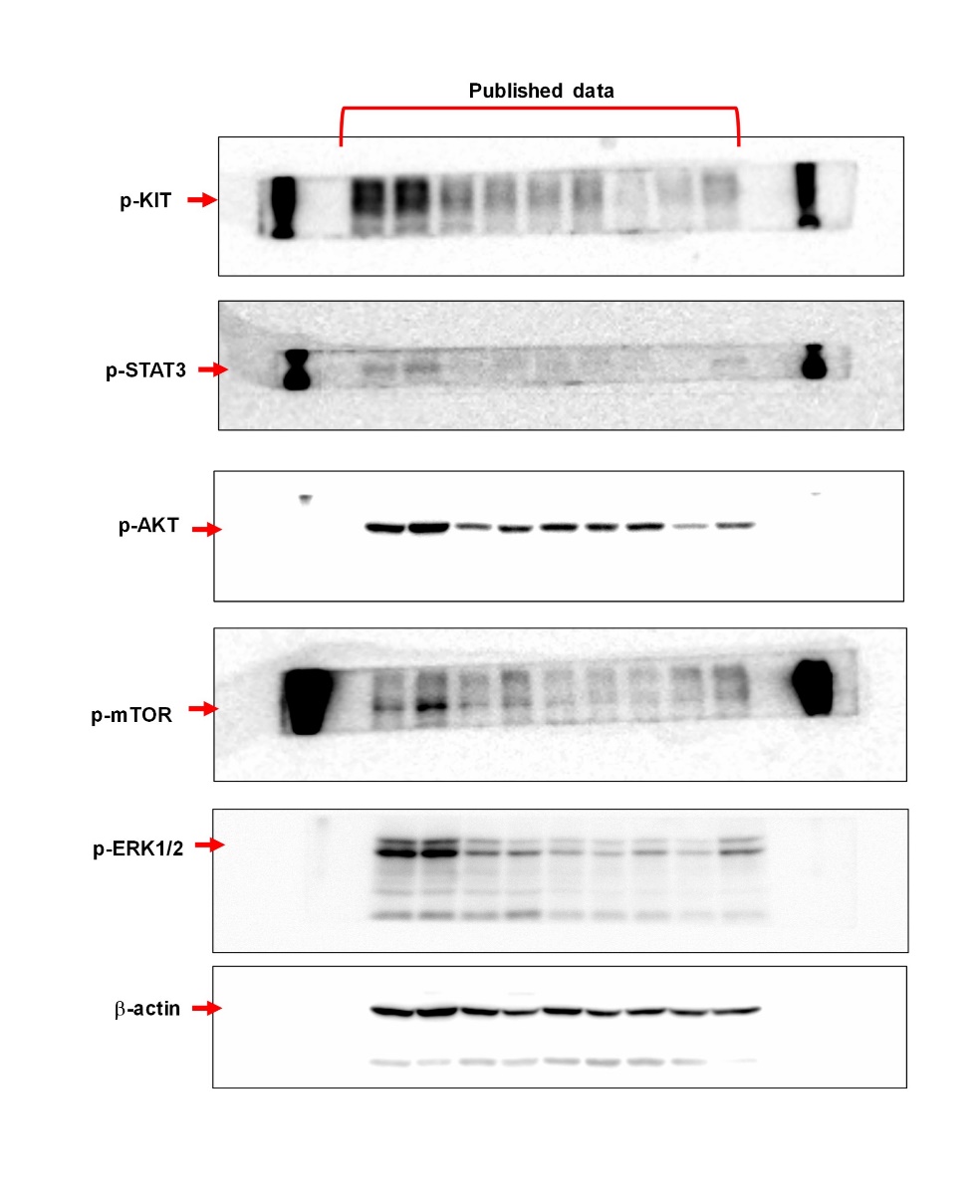
**

**Supplementary Figure 3A.**

**
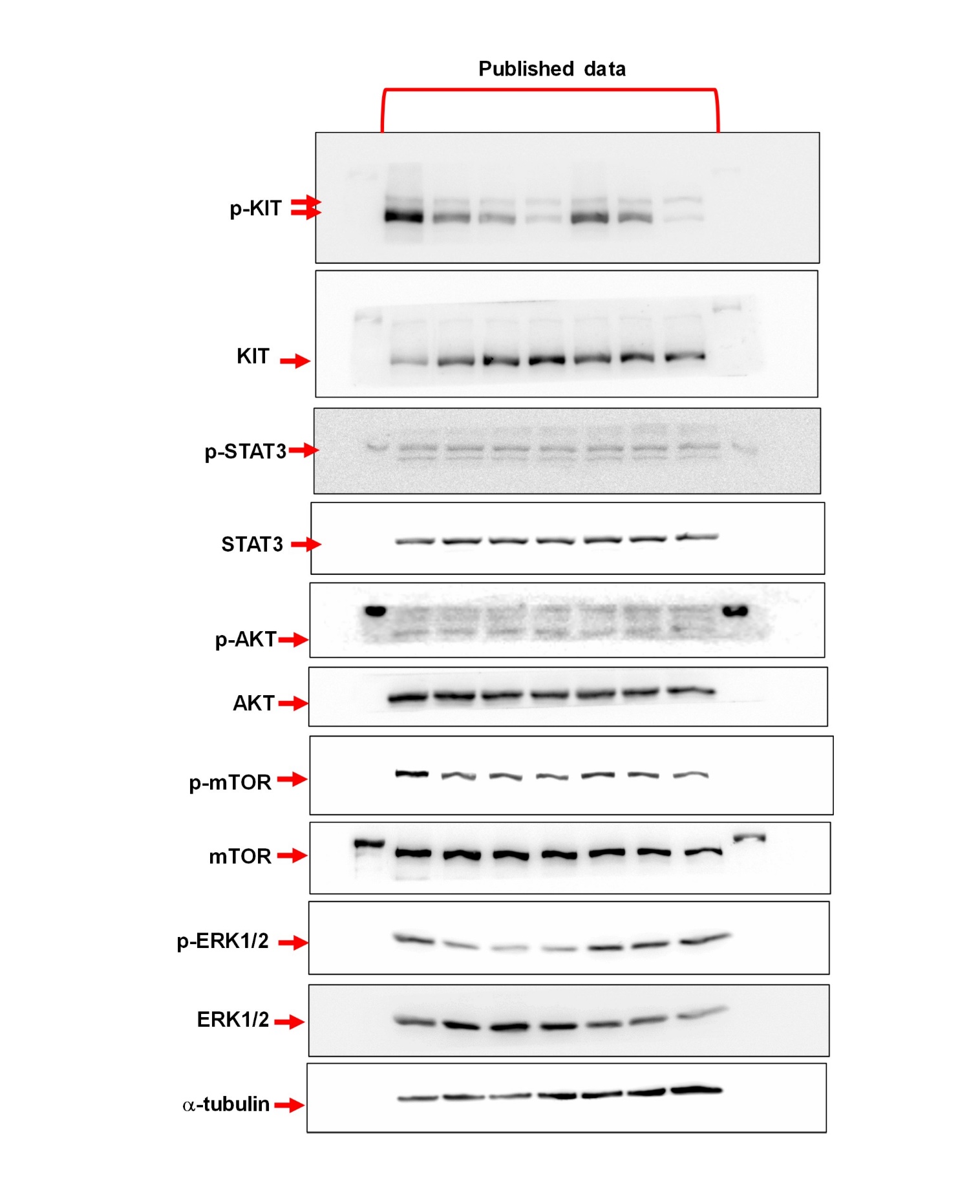
**

**Supplementary Figure 4B.**

**
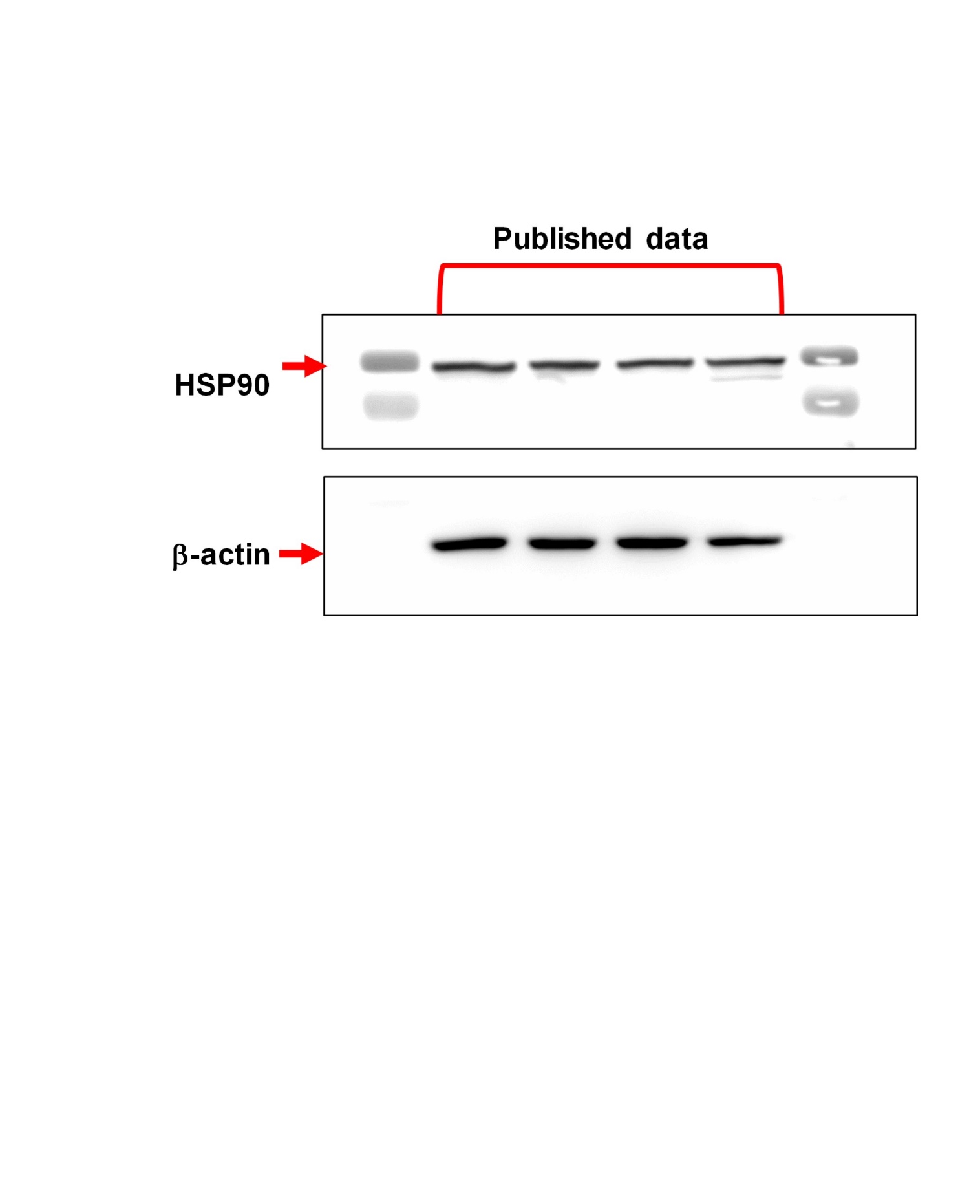
**

**Supplementary Figure 4C.**

**
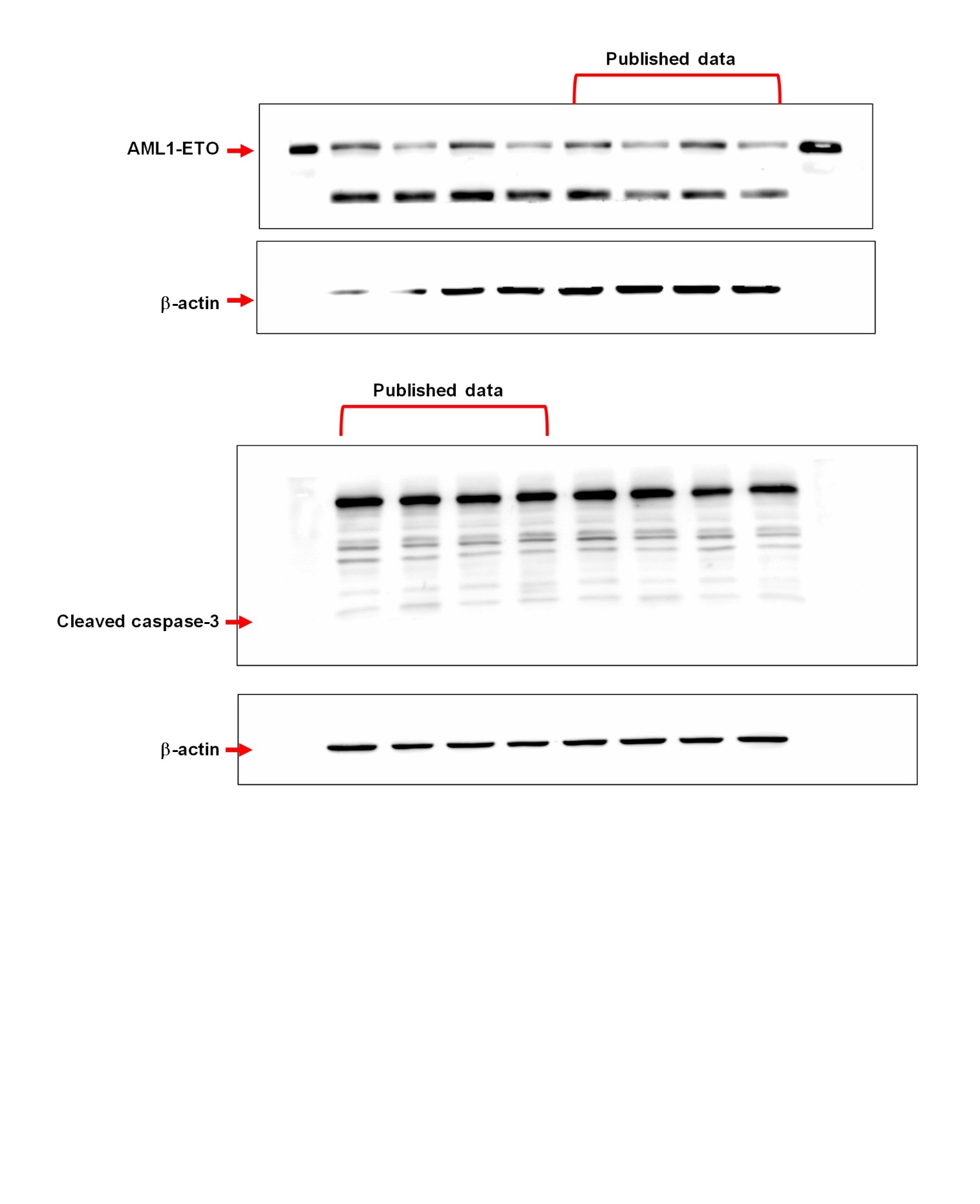
**
